# Transcranial magnetic stimulation of the brain: What is stimulated? – A consensus and critical position paper

**DOI:** 10.1016/j.clinph.2022.04.022

**Published:** 2022-05-18

**Authors:** Hartwig R. Siebner, Klaus Funke, Aman S. Aberra, Andrea Antal, Sven Bestmann, Robert Chen, Joseph Classen, Marco Davare, Vincenzo Di Lazzaro, Peter T. Fox, Mark Hallett, Anke N. Karabanov, Janine Kesselheim, Mikkel M. Beck, Giacomo Koch, David Liebetanz, Sabine Meunier, Carlo Miniussi, Walter Paulus, Angel V. Peterchev, Traian Popa, Michael C. Ridding, Axel Thielscher, Ulf Ziemann, John C. Rothwell, Yoshikazu Ugawa

**Affiliations:** a Danish Research Centre for Magnetic Resonance, Centre for Functional and Diagnostic Imaging and Research, Copenhagen University Hospital Hvidovre, Hvidovre, Denmark; b Department of Neurology, Copenhagen University Hospital Bispebjerg, Copenhagen, Denmark; c Institute for Clinical Medicine, University of Copenhagen, Copenhagen, Denmark; d Department of Neurophysiology, Medical Faculty, Ruhr-University Bochum, Bochum, Germany; e Department of Biomedical Engineering, Duke University, Durham, NC, USA; f Department of Clinical Neurophysiology, University Medical Center, Georg-August-University, Göttingen, Germany; g Department of Clinical and Movement Neurosciences, UCL Queen Square Institute of Neurology, University College London, London, United Kingdom; h Wellcome Centre for Human Neuroimaging, UCL Queen Square Institute of Neurology, University College London, London, United Kingdom; i Krembil Brain Institute, University Health Network and Division of Neurology, University of Toronto, Toronto, Ontario, Canada; j Department of Neurology, University of Leipzig, Leipzig, Germany; k Faculty of Life Sciences and Medicine, King’s College London, London, United Kingdom; l Unit of Neurology, Neurophysiology, Neurobiology, Department of Medicine, Università Campus Bio-Medico di Roma, via Álvaro del Portillo 21, 00128 Rome, Italy; m Research Imaging Institute, University of Texas Health Science Center at San Antonio, San Antonio, TX, United States; n Human Motor Control Section, National Institute of Neurological Disorders and Stroke, National Institutes of Health, Bethesda, MD, USA; o Department of Nutrition and Exercise, University of Copenhagen, Copenhagen, Denmark; p Department of Neuroscience and Rehabilitation, University of Ferrara, Ferrara, Italy; q Non-invasive Brain Stimulation Unit, Laboratorio di NeurologiaClinica e Comportamentale, Fondazione Santa Lucia IRCCS, Rome, Italy; r Sorbonne Université, Faculté de Médecine, INSERM U 1127, CNRS 4 UMR 7225, Institut du Cerveau, F-75013, Paris, France; s Center for Mind/Brain Sciences (CIMeC), University of Trento, Italy; t Cognitive Neuroscience Section, IRCCS Centro San Giovanni di DioFatebenefratelli, Brescia, Italy; u Department of Psychiatry & Behavioral Sciences, School of Medicine, Duke University, Durham, NC, USA; v Department of Electrical & Computer Engineering, Duke University, Durham, NC, USA; w Department of Neurosurgery, School of Medicine, Duke University, Durham, NC, USA; x Center for Neuroprosthetics (CNP) and Brain Mind Institute (BMI), Swiss Federal Institute of Technology (EPFL), Geneva, Switzerland; y Center for Neuroprosthetics (CNP) and Brain Mind Institute (BMI), Swiss Federal Institute of Technology (EPFL Valais), Clinique Romande de Réadaptation, Sion, Switzerland; z University of South Australia, IIMPACT in Health, Adelaide, Australia; aa Department of Health Technology, Technical University of Denmark, Kgs. Lyngby, Denmark; ab Department of Neurology & Stroke, University Tübingen, Tübingen, Germany; ac Hertie Institute for Clinical Brain Research, University Tübingen, Tübingen, Germany; ad Department of Neurology, Fukushima Medical University, Fukushima, Japan; ae Fukushima Global Medical Science Centre, Advanced Clinical Research Centre, Fukushima Medical University, Fukushima, Japan

**Keywords:** Transcranial magnetic stimulation, Motor cortex, Mechanism of action, Physiology

## Abstract

Transcranial (electro)magnetic stimulation (TMS) is currently the method of choice to non-invasively induce neural activity in the human brain. A single transcranial stimulus induces a time-varying electric field in the brain that may evoke action potentials in cortical neurons. The spatial relationship between the locally induced electric field and the stimulated neurons determines axonal depolarization. The induced electric field is influenced by the conductive properties of the tissue compartments and is strongest in the superficial parts of the targeted cortical gyri and underlying white matter. TMS likely targets axons of both excitatory and inhibitory neurons. The propensity of individual axons to fire an action potential in response to TMS depends on their geometry, myelination and spatial relation to the imposed electric field and the physiological state of the neuron. The latter is determined by its transsynaptic dendritic and somatic inputs, intrinsic membrane potential and firing rate. Modeling work suggests that the primary target of TMS is axonal terminals in the crown top and lip regions of cortical gyri. The induced electric field may additionally excite bends of myelinated axons in the juxtacortical white matter below the gyral crown. Neuronal excitation spreads ortho- and antidromically along the stimulated axons and causes secondary excitation of connected neuronal populations within local intracortical microcircuits in the target area. Axonal and transsynaptic spread of excitation also occurs along cortico-cortical and cortico-subcortical connections, impacting on neuronal activity in the targeted network. Both local and remote neural excitation depend critically on the functional state of the stimulated target area and network. TMS also causes substantial direct co-stimulation of the peripheral nervous system. Peripheral co-excitation propagates centrally in auditory and somatosensory networks, but also produces brain responses in other networks subserving multisensory integration, orienting or arousal. The complexity of the response to TMS warrants cautious interpretation of its physiological and behavioural consequences, and a deeper understanding of the mechanistic underpinnings of TMS will be critical for advancing it as a scientific and therapeutic tool.

## 1. Introduction

Since its introduction in 1985 by Barker and colleagues (Barker et al., 1985), the use of transcranial magnetic stimulation (TMS) has revolutionized human brain research, resulting in manifold neurophysiological and therapeutic applications. In contrast to transcranial electrical stimulation (TES), TMS does not directly apply electrical current via electrodes attached to the scalp, but through inductive electromagnetic stimulation. The TMS stimulator passes a short-lasting current through the coil, generating a strong time-varying electromagnetic field perpendicular to the transducing coil, which is placed tangentially on the head. The magnetic field is not attenuated by the tissue surrounding the brain (e.g., skin and bone) and induces a phasic electric field in the targeted tissue. This field can depolarize excitable structures within the brain, e.g. neurons. If the electric field induced by TMS is sufficiently strong to depolarize the membrane potential of a given neuron above a certain threshold, an action potential will be triggered. However, even TMS-induced subthreshold depolarization can have neuronal effects and affect ongoing endogenous activity. Inductive electromagnetic stimulation of a single neuron in the central nervous system is determined by the induced electric field and the morphology and electrophysiological properties of the stimulated neuron at the time of stimulation.

Despite of its widespread use, relatively little is known about how TMS engages the cortical target region and how target engagement propagates within the brain. Basic questions such as which parts of the gyrus, which cell types, and which neuronal compartments are preferentially excited by TMS are still subject to research. The relationship between the biophysical properties of cortical neuronal populations and the efficacy to produce action potentials with TMS in these populations is also still incompletely understood. The same is true for the mechanisms that determine the propagation of the direct TMS-induced neuronal excitation within local cortical circuits and along neural projections to interconnected brain regions.

This consensus paper gives an account on what we currently know and what we still do not know about how TMS “engages” its target, the brain. We summarize the current level of understanding of how TMS targets neural structures in the stimulated area and how the regional induction of action potentials impacts on the cortical target area at the cellular and microcircuit level. We also discuss how regional neural excitation of the stimulated cortex influences neural function of remote brain areas. Plasticity-inducing effects of TMS are not covered. We first synthesize some general considerations about TMS and its underlying mechanisms in section 2. We then focus specifically on how TMS stimulates the primary motor cortex (M1) in section 3. We finally review key insights into the mechanism of action of TMS that have been gathered with TMS targeting other brain regions than the M1 in section 4.

## 2. General considerations

### 2.1. Physiological features and their mechanistic implications

#### The TMS-induced electric field directly interacts with axons in the targeted cortex.

The direct neural response to the TMS-induced electric field is complex, involving a mixture of neuronal populations (Aberra et al., 2020). Invasive recordings from rodent motor cortex as well as from the descending corticospinal tract in humans show that a single TMS pulse evokes a cascade of high-frequency synaptic activity in the stimulated motor cortex (see section 3.1.1 for detailed discussion) (Di Lazzaro and Rothwell, 2014; Li et al., 2017). Regional neuronal excitation outlasts the stimulus by several milliseconds and depends on the orientation of the induced electric field (Di Lazzaro and Rothwell, 2014; Li et al., 2017). This begs the question what is the primary neural target of the initial current pulse? Many studies have addressed this question using TMS of the hand representation of the M1 (M1-HAND), because TMS of M1-HAND can readily produce a motor response that can easily be recorded with surface electrodes from the responding muscles in the contralateral hand (Groppa et al., 2012a). These studies will be discussed in detail in the section on TMS of the M1 (section 3), but we summarize some fundamental features already here because of their general relevance.

#### TMS stimulates neurons in the brain through depolarization of myelinated axons.

By systematically varying the intensity and duration of the TMS pulse, one can derive a curve, which delineates a threshold function for evoking a motor evoked potential (MEP). This curve reflects the membrane excitability of the neural structures that are stimulated with TMS and can be mathematically described by its strength–duration (S-D) properties and the closely related neurophysiological concepts of chronaxie and rheobase (Geddes and Bourland, 1985). Rheobase describes the minimum current amplitude (of infinite duration) that leads to threshold depolarization, whereas chronaxie is the minimum time required for a current at double the strength of the rheobase to initiate an action potential. Chronaxie is equivalent to the S-D time constant. Measurements of the relationship between the minimal intensity and minimal duration of a TMS pulse to evoke a MEP over M1-HAND show that TMS is likely to activate axons rather than neuronal cell bodies (Barker et al., 1991; D’Ostilio et al., 2016; Hannah and Rothwell, 2017; Peterchev et al., 2013). The cell soma has a much longer (membrane) time constant and a higher excitation threshold than the axon (Frank and Fuortes, 1956; Nowak and Bullier, 1998). Experimentally, this can be tested by using controllable TMS devices that can change the duration as well as the amplitude of the stimulus pulse (D’Ostilio et al., 2016; Halawa et al., 2019; Hannah and Rothwell, 2017). However, the range of available pulse durations in current controllable TMS devices is relatively small (30–150 μs) compared with the ranges usually employed when making measurements with a standard electrical pulse (up to 1000 μs). In addition, a TMS pulse is never a perfect square wave and is always followed by a reverse phase, resulting in no net transfer of charge. The outcome is that the S-D time constant measured in this way is not equal to that measured with conventional square wave electrical pulses. Nevertheless, when the same magnetic stimulator is used to stimulate peripheral nerves and the motor cortex, the calculated time constants of both of them are similar (i.e., around 150–300 μs), suggesting that similar structures, namely large myelinated axons, are likely to be one of the prime targets of TMS in either case (Barker et al., 1991; Peterchev et al., 2013).

#### Which axonal structure is primarily stimulated by the induced electric field?

**Fig. 1.**
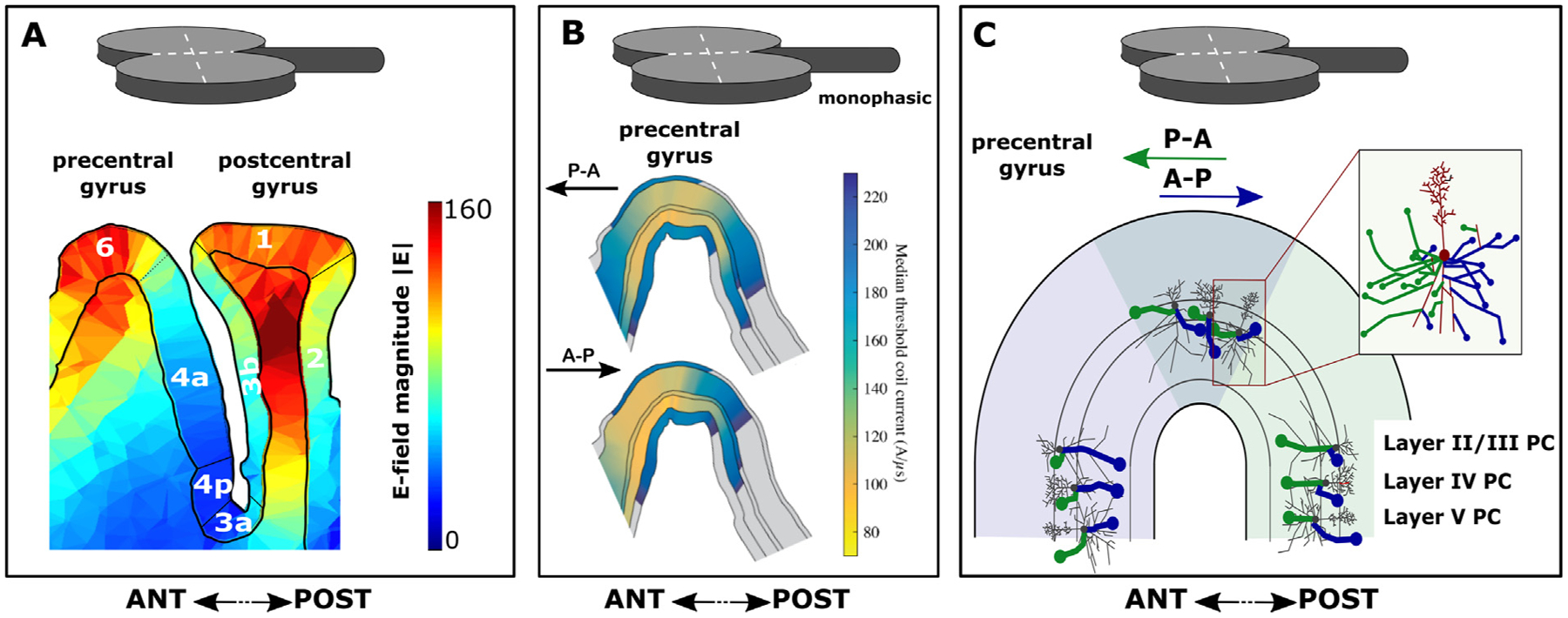
Sagittal view on the pre and postcentral gyrus illustrating key biophysical features of transcranial magnetic stimulation (TMS). The sagittal slice cuts through the motor hand knob which hosts the precentral motor hand representation. **Panel A.** Spatial pattern of the electric field magnitude (|E|) induced by TMS in both precentral and postcentral gyrus (generated with SimNIBS software). Note that the highest field strengths are obtained in the crowns of the pre- and postcentral gyri. The illustration also shows that significant “hot spots” may arise in subcortical white matter, although the activation threshold there is likely to be different than in the gray matter due to differences in the represented neural elements. The numbers indicate the various cyto-architectonically defined cortical areas according to Brodmann. **Panel B. Layer-specific distribution of activation thresholds in relation to induced current direction in the hand knob of the pre-central gyrus.** Shown are median thresholds for layers 1–6 on analysis plane through pre-central gyrus, parallel to coil handle and near coil center for monophasic stimulation with posterior-anterior (P-A) and anterior-posterior (A-P) current directions. The thresholds were simulated with a multi-scale model coupling electric field distribution from Panel A to morphologically realistic cortical neuron models in NEURON software. Modified from Aberra and colleagues (2020) with permission. **Panel C. Direction-specific depolarization of axon terminals illustrated for pyramidal cells (PC) in cortical layers II/III, IV and V.** Pyramidal cells, including their axonal arborization, are “projected” into the anterior (light blue) and posterior part (light green) of the precentral gyrus, forming the posterior wall of the precentral gyrus or anterior wall of the central sulcus, respectively. The same cells are also projected onto the crown of the precentral gyrus (grey area). Depending on the induced current direction in the precentral gyrus, different terminals of axonal branches are primarily depolarized by the TMS-induced electric field. These axons are highlighted as bold blue and green lines according to induced current directions. Axon branches susceptible to a posterior-anterior (P-A) current direction in the gyrus are labeled in blue and axon branches susceptible to anterior-posterior (A-P) current direction are labelled in green. The dendritic tree, soma and axonal branches perpendicular to the P-A and A-P directions are labeled in grey and red color. From a biophysical modeling perspective, the axon terminal mechanism of action potential induction illustrated in this panel is a key mechanism by which TMS induces action potentials, but it does not exclude additional mechanisms (e.g. excitation at axonal bends), especially at high intensities of stimulation. The illustration is inspired by results from the multi-scale model depicted in Panel B. Please note that the real size of the TMS coil is much larger. ANT: anterior, POST: posterior.

**Fig. 2.**
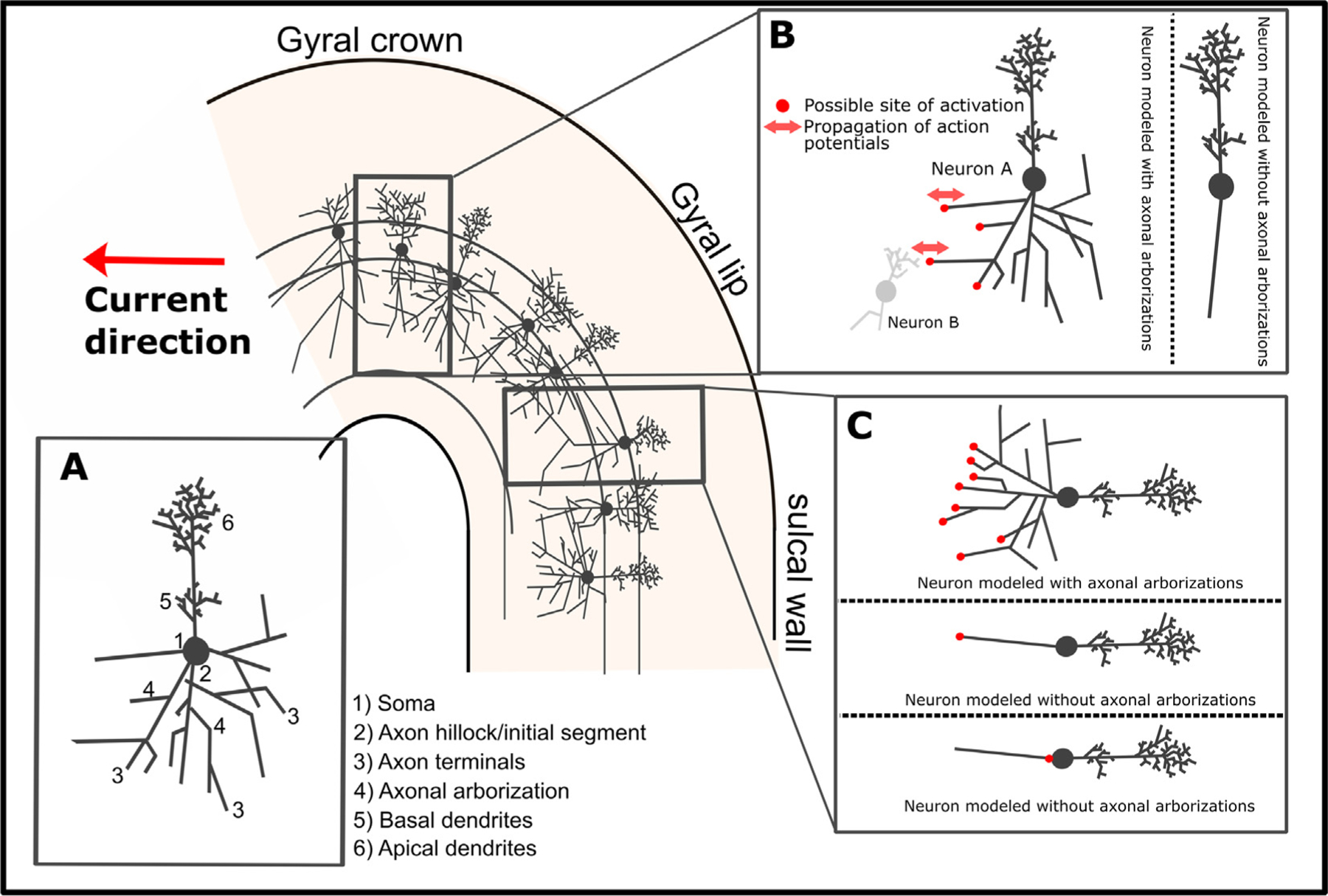
Theoretical accounts for the site of activation for transcranial magnetic stimulation (TMS) in the precentral gyrus. The figure displays a sagittal slice through the motor hand knob of the precentral gyrus with pyramidal cells occupying layer II/III. **Inset A. Drawing of a single pyramidal cell.** Displays a drawing of a single pyramidal cell with key anatomical features highlighted. **Inset B. Activation of pyramidal cell in the crown or lip region.** The panel depicts a pyramidal cell (Neuron A) located in the crown of the precentral gyrus. Possible sites of activation with a monophasic current (posterior-to-anterior direction) are highlighted in red for pyramidal cells modelled with and without axonal arborizations. Note that the axon terminals constitute primary targets when the pyramidal cell is modeled with arborizations. Neural excitation at the axon terminals will lead to propagation of action potentials in both orthodromic and antidromic directions. The orthodromic propagation leads to transsynaptic effects in downstream neurons (e.g. Neuron B). In contrast, when the neuron is modelled without axonal arborizations, activation is unlikely to take place in the crown region of the gyrus. This is in accordance with the phenomenological cortical column cosine theory (Fox et al., 2004) and demonstrated via modeling in Aberra et al., (2020). **Inset C. Activation of a pyramidal cell in the lip region of the gyrus or in the sulcal wall.** The panel shows a pyramidal cell located at the border between the lip region and the sulcal wall of the precentral gyrus. Possible sites of activation with a monophasic current (posterior-to-anterior current direction) are highlighted in red for cells modelled with and without axonal arborizations. Please note that activation at e.g. the axon terminal or the axon hillock can lead to both orthodromic and antidromic propagation of action potentials. The orthodromic activation will lead to transsynaptic effects. Induction of action potentials at the axon terminals (or axon hillocks, although this is less plausible from a biophysical modeling perspective) provides a key mechanism through which TMS exerts its neuronal effects. This does not, however, preclude other potential sites of activation such as excitation at axonal bends as discussed in the text.

Cable theory provides a theoretical framework to understand how the TMS-induced electric field polarizes and activates axons. Axons can be stimulated by electric field gradients along their length, if the gradients are strong enough to trigger an action potential. An electric field gradient along the axon can occur due to spatial changes in the induced electric field or due to changes in the axon geometry (Tranchina and Nicholson, 1986) The former mechanism polarizes the axon proportional to the first spatial derivative of the electric field, whereas the latter mechanism polarizes the axon proportional to the electric field magnitude at geometrical discontinuities (e.g. terminations, bifurcations, and bends). The first mechanism is less relevant for TMS, as the spatial gradient of the TMS-induced electric field is typically negligible at the scale of cortical neurons; thus, the electric field is often treated as quasi-uniform (Bikson et al., 2013). These activation mechanisms were demonstrated with magnetic stimulation of *in vitro* peripheral nerves, which found that long, straight axons are activated at the site of peak electric field gradient. Introducing bends or terminations (e.g. cut ends) shifted excitation to these locations, where the threshold was inversely proportional to the electric field magnitude (Maccabee et al., 1993). The situation in the brain is much more complex. This is illustrated in Fig. 1C and Fig. 2 for pyramidal cells occupying layer II/III. While there is consensus that the biophysical and geometric properties of the axons determine which axonal structures are effectively depolarized by the electrical current, debate still remains about the exact site of activation by TMS.

Based on these considerations, several candidate sites of excitation by TMS have been identified, including axon terminations, changes in diameter (e.g. soma–axon hillock), and bends (e.g. corticofugal axons curving into subcortical white matter) (Roth, 1994). Within cortical grey matter, realistic models of cortical neurons that include axonal arborization identified the terminals of axon collaterals, aligned to the local electric field direction, as the primary site of activation (Aberra et al., 2018; Salvador et al., 2011). According to these models, the TMS-induced current depolarizes axon terminals when the current is running in parallel to the distal axon branch and is directed towards the axon terminal, as illustrated in Fig. 1C and Fig. 2BC (Aberra et al., 2018). Activation thresholds of these axon terminals were significantly reduced by the presence of myelination, which reduces the membrane capacitance and, consequently, the S-D time constant.

It has also been argued that the axon initial segment is primarily activated by TMS after somatic depolarization (Fig. 2C), because the diameter of the neuron abruptly changes at the transition from soma to axon hillock (Pashut et al., 2011). However, the results of these early modeling studies may have been due to implementation errors resulting in an artefactual current source in the soma (Aberra et al., 2020). Preferential activation of axon terminals, rather than initial segments, is also consistent with the finding that the threshold to evoke the earliest corticospinal wave (I_1_) by TMS is relatively insensitive to voluntary contraction, GABA-agonists, or the inhibitory paired-pulse paradigm, short interval intracortical inhibition (SICI) (Di Lazzaro and Rothwell, 2014). Since fluctuations in membrane potential from synaptic inputs attenuate significantly with distance from the soma, these observations suggest TMS-evoked action potentials are initiated in the distal axon.

TMS at low intensities for most coil configurations is known to directly activate axons in cortical grey matter (Di Lazzaro and Rothwell, 2014), but juxtacortical excitation of axonal bendings in subcortical white matter is another possible mechanism, which has been demonstrated in several models as well (Geeter et al., 2016; Gomez-Tames et al., 2020; Goodwin and Butson, 2015; Salvador et al., 2011). This may include juxtacortical bendings of cortico–cortical axons (e.g. U-fibers) or descending pyramidal axons (e.g. projecting to the pyramidal tract) in the superficial white matter underlying the gyral crown. The hypotheses that TMS primarily excites intracortical axon terminals or axonal bends in juxtracortical white matter are not mutually exclusive. Based on passive cable theory, one can estimate relative difference in coupling to the E-field, or with active models, one can predict threshold differences. However, it is difficult to predict exact differences in relative thresholds for excitation of intracortical axon terminals versus axonal bends in juxtacortical white matter. These axonal segments would constitute very different and heterogenous populations (white matter fibers vs. intracortical axon terminals) with potentially different electrophysiological properties.

In summary, there are several accounts of where the TMS-induced electric field primarily excites the intracortical axons. The notion that TMS primarily causes intracortical excitation of myelinated axons at their terminals is biophysically the most plausible account. Regardless of which of the various accounts is correct, they all predict that the efficacy of TMS to depolarize the axonal elements critically depends on the orientation of the axonal element relative to the direction of the induced electric field and the magnitude of the electric field. It is also possible that multiple mechanisms play a role in TMS excitation depending on the stimulation parameters, including intensity, direction, and pulse waveform.

#### Direction-specific effects play an important role for axonal depolarization.

The directional sensitivity of stimulation sites is particularly evident when a figure-of-eight coil and a “monophasic” pulse configuration (meaning a brief, high amplitude electric field phase in one direction followed by a longer, low amplitude phase in the opposite direction) is used for TMS. Direction-specific effects are clearly evident when stimulating the M1-HAND in the precentral gyrus with a figure-of-eight coil and a monophasic pulse configuration (see section 3.1 for detailed discussion). Generally, TMS is most effective at evoking a MEP if the dominant induced tissue current has a posterior-to-anterior (P-A) direction and has a perpendicular orientation relative to the wall of the central sulcus (Fig. 1). A TMS pulse inducing a P-A current in the precentral gyrus will evoke MEPs in hand muscles that have a lower threshold and shorter latency compared to a pulse of equal stimulus strength that induces an anterior-to-posterior (A-P) directed current (Mills et al., 1992). In accordance with the differences in MEP latency, epidural recordings of the descending corticospinal activity show that P-A oriented currents evoke preferentially early volleys, known as indirect waves (I-waves), including the I_1_-wave generated by monosynaptic inputs to corticospinal neurons, whereas A-P oriented currents in M1-HAND preferentially evoke later I-waves (Di Lazzaro and Rothwell, 2014; Sakai et al., 1997). Biophysical modeling suggests that reversing the current direction in the precentral gyrus from P–A to A–P leads to an anterior spatial shift in preferential direct activation of neural populations in the precentral crown, particularly the pyramidal cells in L2/3 and L5 and the large basket cell inhibitory interneurons (Aberra et al., 2020) (Fig. 1BC). This may induce differences in the time it takes for the action potentials generated at the site of primary stimulation in the top and lip regions of the precentral crown to transsynaptically excite those corticomotoneuronal pyramidal cells that are buried in the sulcal wall and make monosynaptic connections onto spinal alpha-motoneurons (i.e., corticomotoneuronal cells). Electrophysiological measurements suggest that direction-specific transsynaptic inputs to the corticomotoneuronal cells also have slightly different S-D time constants (D’Ostilio et al., 2016). Such direction-specific effects are also present when using biphasic pulse configurations, though to a lesser degree (Aberra et al., 2020; Lang et al., 2006).

The direction dependency of TMS-evoked axonal depolarization has general implications for the clinical and scientific use of TMS. Different TMS-induced current directions will result in preferential targeting of spatially distinct population of neurons, even when the location of the peak induced electric field is matched (Aberra et al., 2020). These differences in the primary site of neural excitation may result in differences at the neurophysiological and behavioral level. The direction-specific effects may be most prominent at stimulation intensities that are slightly above excitation threshold and may become less specific at higher intensities of stimulation, when the induced tissue current results in a less selective activation of neuronal elements in the targeted cortex.

In addition to the direction of the induced current, the shape and width of the TMS pulse may also affect which neuronal circuits that are activated by a TMS pulse, as shown using controllable TMS devices that allow some flexibility in terms of pulse width and shape. For example, manipulating the pulse-width may lead to a recruitment of distinct neuronal populations with different S-D constants (D’Ostilio et al., 2016) that are differently associated with cutaneous and proprioceptive afferent inputs as revealed by conditioning TMS protocols (Hannah and Rothwell, 2017). These effects also seem to interact with current direction, highlighting the intricate nature of the physiology of TMS.

#### TMS effectively excites inhibitory interneurons in the stimulated brain area.

Converging neurophysiological evidence for TMS-induced excitation of intracortical inhibitory circuits stems from paired-pulse TMS targeting the M1 (see section 3.5). A sub-threshold conditioning TMS pulse inhibits the motor output evoked by a subsequent supra-threshold test stimulus (Bestmann et al., 2004), resulting, for example, in short-interval intracortical inhibition (SICI) (Kujirai et al., 1993). Single-pulse TMS of the M1 can also reveal intracortical inhibition, producing a post-excitatory silent period in a pre-contracted target muscle (Wilson et al., 1993). The inhibitory intracortical circuits respond to TMS at stimulation intensities that are well below the threshold to evoke a MEP in relaxed hand muscles (Davey et al., 1994; Ziemann et al., 1996d). Cortical inhibitory interneurons release the neurotransmitter γ-aminobutyric acid (GABA) onto excitatory and other inhibitory neurons. It is commonly believed that the inhibitory effects of TMS are mediated by inhibitory interneurons through a GABA-ergic suppression of excitatory TMS effects, although “shunting inhibition” caused by an activity-driven increase in transmembrane conductance has been suggested as an alternative mechanism (Paulus and Rothwell, 2016). In paired-pulse TMS paradigms that use short inter-stimulus intervals, neural excitation of the first conditioning pulse may acutely increase membrane conductance in the dendritic tree of cortical pyramidal cells. This “leaky” membrane conductance may reduce the transsynaptic current flow from the dendritic tree to the soma induced by the second TMS pulse (Paulus and Rothwell, 2016).

#### TMS-induced neuronal stimulation propagates along axons and synapses.

The action potentials induced directly by the TMS-induced electric field travel along the axons in the anterograde (orthodromic) and retrograde (antidromic) direction, inducing forward and backward information flow within the stimulated pathway (Fig. 2). An anterograde axonal propagation of excitation is likely to make a major contribution to the neurobiological network effects of TMS due to transsynaptic excitation along connected chains of neurons (Pashut et al., 2014; Rotem and Moses, 2006, 2008). Action potentials propagate transsynaptically, producing local excitation or inhibition of intracortical circuits directly within the targeted cortex as well as remote effects of interconnected cortical and subcortical areas. The best-known example is TMS of the M1 which gives rise to a somatotopically specific motor response, once stimulus intensity surpasses the cortical motor threshold (MT).

#### State dependency is an inherent feature of TMS.

Both local transsynaptic excitation of neural structures in the targeted cortex as well as the transsynaptic propagation of excitation to remote brain regions are modulated by state-dependent factors, including ongoing neuronal firing rate and intrinsic excitability. The motor response to TMS of M1 is influenced by the “motor state” at the time of stimulation: Using identical stimulation settings, the MEP amplitude obtained at rest can be facilitated or suppressed by motor imagery involving the target muscle (Kasai et al., 1997; Sohn et al., 2003). Depending on the state of perceptual adaptation, an identical TMS pulse given to visual cortex can induce differential effects on spatially overlapping neuronal populations in the stimulated cortex and thus evoke different types of TMS-evoked phosphenes (Silvanto et al., 2007).

Electroencephalographic (EEG) recordings of the TMS-evoked EEG potentials (TEPs) during non-rapid eye movement (NREM) sleep have also convincingly demonstrated that the brain state at the time of stimulation determines the brain’s response to TMS (Bergmann et al., 2012; Massimini et al., 2005). NREM sleep enhances the short-latency local response to TMS, but shows a marked attenuation of the propagation of excitation to remote cortical sites that can be observed during wakefulness (Massimini et al., 2005). Furthermore, MEPs and TEPs have higher amplitudes when the TMS pulse is given during up-states than during down-states of slow wave oscillations (Bergmann et al., 2012). These examples show that physiological and behavioral effects of TMS depend not only on the TMS settings (extrinsic factors) but also on the functional properties of the stimulated brain network (intrinsic factors). This explains the increasing interest in online “state markers” that can be used to inform TMS in order to increase the reliability and efficacy of TMS.

#### TMS excites multiple sites in the peripheral nervous system (Fig. 3).

The induced electric field does not only excite neuronal structures in the central nervous system. Peripheral co-stimulation of sensory and motor axons in the face or neck region and intracranial sensory and motor axons at the base of the skull may also be effectively excited by TMS (Schmid et al., 1995). TMS induces eddy current in the cerebrospinal fluid, which can lead to excitation of all motor (and probably also sensory) fibers of the facial nerve close to the foramen ovale (Schmid et al., 1992, 1995). Foraminal excitation of myelinated motor axons of the facial nerve occurs already at low stimulus intensities with threshold intensities ranging between 20 and 40% of maximal stimulator output, when using a standard round coil and a Magstim 200 device (Schmid et al., 1995). Foraminal motor responses of the facial nerve showed orientation dependency and were readily elicited at many lateral stimulation positions across the scalp, when the center of a round stimulation coil was positioned at electrode positions C3 (the approximate location of M1-HAND), P3 and T3 of the international 10–20 system for EEG electrode placement (Schmid et al., 1995). The trigeminal nerve fibers can also be excited by TMS at their proximal segment distal to the root entry zone, but the threshold for stimulation is higher and the scalp position of TMS is more critical for effective stimulation (Schmid et al., 1995). Foraminal co-stimulation of cranial nerve fibers poses a methodological challenge in terms of experimental control, because this type of peripheral co-stimulation will occur at many lateral fronto-temporal cortical target sites and cannot be matched by peripheral electrical stimulation.

**Fig. 3.**
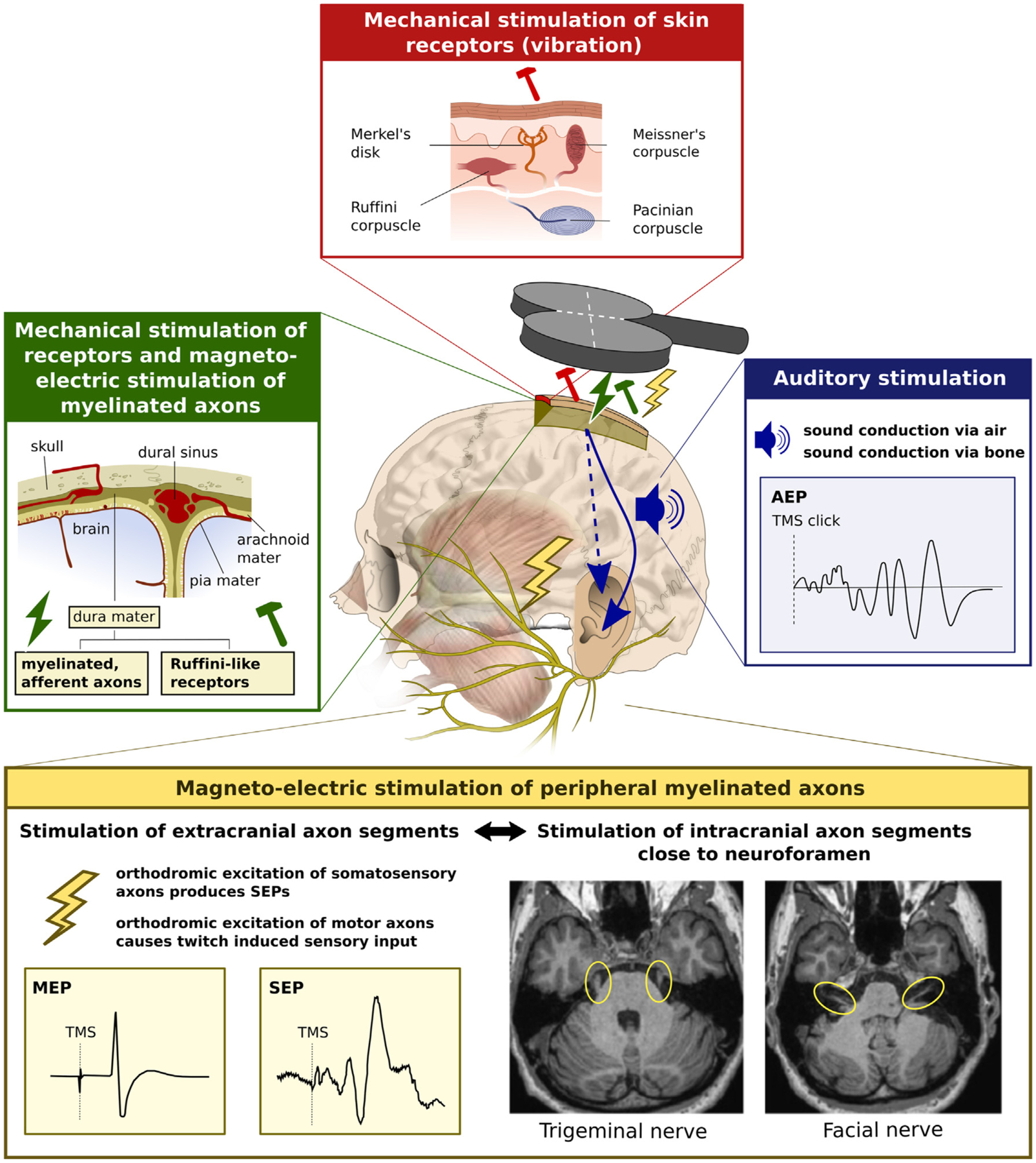
Multiple sites of peripheral co-stimulation. The figure summarizes peripheral sensory receptors and axons that can be excited by transcranial magnetic stimulation (TMS). **Blue box.** Auditory stimulation by the loud, high frequency click sound produced in the coil and cable during discharge, causing auditory evoked potentials (AEP) in the EEG. **Yellow box.** Somatosensory stimulation of peripheral sensory and motor axons (i.e., peripheral branches of the facial, trigeminal or occipital nerve) give rise to cortical somatosensory potentials (SEPs). Excitation of peripheral motor nerves lead to sensory input caused by the evoked muscle twitches. Twitch-induced sensory input also occurs, when TMS of motor cortex produces motor evoked potentials (MEP). In addition, the proximal segments of the facial and trigeminal nerves can be effectively excited by TMS at many scalp sites, even within the commonly used range of stimulus intensities. **Green box.** Somatosensory stimulation may arise from magneto-electric stimulation of afferent myelinated nerve fibers or mechanical stimulation of unencapsulated Ruffini-like receptors in the dura mater. **Red box.** The skin contains various receptors responding to coil-induced tonic pressure or TMS-induced coil vibration (Meissner’s corpuscles, Merkel’s disks and Pacinian corpuscles) and stretch due to coil movement (Ruffini corpuscles).

There are several other sources of somatosensory co-stimulation, including the excitation of mechanoreceptors due to TMS-induced vibration, and reafferent somatosensory stimulation caused by TMS-evoked muscle twitches (Fig. 3). The dura mater is another potential site of peripheral excitation. The parasagittal dura mater contains Ruffini-like mechanoreceptors as well as myelinated fast-conducting A-beta fibers (Lv et al., 2014). The former may be excited by mechanic vibrations, while the latter may be excited by the induced electric field. Future studies need to clarify whether peripheral somatosensory co-stimulation of the dura mater is relevant and how much it depends on coil position and orientation. There is circumstantial evidence in stroke patients that these inputs are unlikely to play a significant role. Using auditory masking and a foam layer between coil and scalp, Sarasso et al. (2020) demonstrated that focal TMS of the lesioned cortex often failed to produce TEPs, while perilesional and contralesional TMS consistently elicited TEPs.

In addition to co-stimulation of peripheral somatosensory and motor fibers, TMS also produces a loud click in the coil case, which leads to auditory stimulation via air and bone conduction (Koponen et al., 2020). The vibration of the skull can be expected to be a critical confounding factor that may influence TMS-induced brain activation, because unnoticed cochlear fluid vibration has been reported to have prominent effects on brain activation caused by ultrasound in animals (Guo et al., 2018).

The multiple peripheral effects of TMS result in substantial “off-target” brain stimulation. Multisensory peripheral co-stimulation is inherent to TMS and needs to be taken into consideration when designing a TMS study because it may conflate or obscure the true transcranial effects of TMS on functional brain activity and render the causal interpretation of neurophysiological, behavioral or therapeutic effects ambiguous (Conde et al., 2019). Of note, the sensation of stimulation experienced by the subjects is significantly affected by the width the TMS pulse. This was observed in a study in which different TMS pulse width were tested and the pulse amplitude was adjusted according to the motor threshold, hence producing the same or similar cortical activation (Peterchev et al., 2017). This difference in subjective experience may result from a combination of direct scalp nerve/muscle stimulation, coil vibration, and sound difference. In any case, the effects of TMS pulse width on subject’s sensory experience appear to follow a different relationship than the effects of pulse width on cortical stimulation (Peterchev et al., 2017).

Peripheral co-stimulation effects should be assessed and reported in detail. The magnitude of peripheral co-stimulation should be minimized, and its impact should be masked or matched by experimental control condition as much as possible (Belardinelli et al., 2019; Siebner et al., 2019). There are a few exceptions when this may not be necessary, for instance when measuring short-latency interaction in the brain with paired-pulse TMS at very short interstimulus intervals. However, even in these cases, the subject’s ability to anticipate the timing of TMS and peripheral co-stimulation may condition the brain response to TMS (Bonnesen et al., 2022).

### 2.2. Biophysical features and their mechanistic implications

#### TMS-induced neurostimulation is biased towards the superficial parts of hemispheric neocortex.

When targeting a cortical area located on the lateral surface of the hemisphere, cortical patches located in the gyral crown will always be exposed to a stronger electric field than cortical patches located in the wall of the gyrus or at the fundus of a cortical sulcus (Fig. 1). This is due to the limited depth penetration of TMS caused by a rapid decay of the induced electric field with increasing distance from the coil. When neglecting the impact of the head as a volume conductor, the electric field decay of circular coils is approximately inverse quadratic, similar to the electric field of a magnetic dipole, and the electric field decay of figure-8 coils is approximately inverse cubic (Deng et al., 2013; Gomez et al., 2018; Heller and van Hulsteyn, 1992). The actual decay inside the head is even steeper as the electric field approaches zero in the brain center. It would be exactly zero if the head was a perfect sphere (Deng et al., 2013; Gomez et al., 2018; Heller and van Hulsteyn, 1992). Depth penetration can be increased by increasing stimulus intensity or by using larger coils with an optimized configuration, but these modifications will always result in a substantially higher and less focal electric field in superficial cortical structures, precluding selective “deep” TMS (Deng et al., 2013, 2014; Gomez et al., 2018; Heller and van Hulsteyn, 1992). At best, even for very large and practically infeasible coils with a uniform magnetic field, the electric field decays linearly with depth, still reaching zero at the head center while also exposing the brain to an extremely non-focal stimulation (Deng et al., 2014).

The limited depth penetration has important practical implications for the use of TMS. When using a stimulation intensity that is sufficient to effectively target the fundus region of a cortical gyrus, TMS will always result in a stronger concurrent stimulation of more superficial parts of the gyrus, such as the top and lip regions in the crown. A depth gradient in terms of effective stimulation also exists when stimulating cortical areas in the mesial wall. When targeting mesial cortical areas such as SMA or primary motor leg area, the superficial cortex close to the midline of the hemispheric convexity will be exposed to a stronger electrical field than the mesial cortical areas located in the interhemispheric fissure. Another implication is that TMS is unable to directly target deep structures of the cerebral hemispheres such as cingulate cortex, medial parts of the temporal lobes, cortex around the insular fissure, thalamus and basal ganglia. However, these deep brain structures may be effectively stimulated by a spread of excitation from effectively stimulated superficial cortical areas to the cingulate cortex via white-matter connections. Regarding depth penetration, a notable exception are local electric field maxima caused by non-uniformities in the heterogeneous tissues (Roth, 1994). These local field maxima might cause remote spots of effective axonal stimulation. A relevant example is remote stimulation of the spinal or cranial nerves as they pass through foramina in the vertebrae or the base of the skull, resulting in effective stimulation of the proximal nerve segments (Groppa et al., 2012a; Ugawa et al., 1989) (see also section 2.1). It should be noted, though, that the electric field attenuates so rapidly in depth within the brain that perturbations of the field strength due to tissue inhomogeneities do not provide a substantial advantage in terms of depth of stimulation.

#### What constitutes the primary cellular target of TMS in terms of neuronal excitation?

The prevailing view is that TMS activates primarily myelinated cortical axons at their bends, bifurcations or terminations (Roth, 1994; Roth and Basser, 1990), with lowest thresholds likely occurring at terminations, as discussed in section 2.1. Dendrites are less responsive to the induced electric field, because of their higher chronaxie value (or S-D time constant) relative to axons (Stern et al., 2015). Myelinated axons exist both in grey and white matter, rendering both tissues excitable to TMS, provided a sufficiently strong local electric field. Out-projecting axons originating from pyramidal cells as well as incoming axonal projections, e.g. of cortico–cortical projection neurons, might also be neural target structures. Generally, thicker myelinated axons are more excitable than thin unmyelinated axonal fibers (Reilly, 1989) or the cell soma (Nowak and Bullier, 1998). As already mentioned in section 2.1, MEP-based measurements of S-D curves demonstrated that TMS of M1 excites neural structures with membrane time constant of 150–300 μs, matching the membrane time constants of myelinated peripheral nerves (Barker et al., 1991; D’Ostilio et al., 2016; Peterchev et al., 2013).

#### The excitability of neural structures such as myelinated axons is influenced by their orientation relative to the electric field.

This notion was corroborated by *in vitro* studies combining inductive magnetic stimulation with electrophysiological recordings from nerve preparations, cell cultures or rodent brain slices (Pashut et al., 2014; Rotem and Moses, 2006, 2008). Axon bends are most easily excited when the axon is parallel to the induced field at one side of the bend while being perpendicular at the other side (Amassian et al., 1992; Maccabee et al., 1993; Maccabee et al., 1998). This suggests bends of myelinated axons in the juxtacortical white matter are susceptible to excitation by the TMS-induced electric field. This mechanism may be particularly relevant for axonal bends of cortico-cortical or cortico-subcortical projection neurons that originate from the gyral crown-lip region.

#### Other axonal segments can also form local spots that display low excitation threshold.

In addition to bends, axonal segments which display a change in diameter or myelination and axonal endings (i.e., axon terminals) may be preferentially activated by TMS (Maccabee et al., 1998; Nagarajan et al., 1997; Roth, 1994). The importance of axon terminals was emphasized in biophysical modeling studies that used cell models with realistic (Aberra et al., 2018; Rahman et al., 2013) and idealized axonal arborizations (Aberra et al., 2018; Rahman et al., 2013; Salvador et al., 2011).

The ability of TMS to excite distal elements of the axonal tree broadens the range of potential intracortical target sites: TMS may effectively stimulate medium or long-range cortico-cortical axons that project into the targeted area or short-range connections formed by local axon collaterals. Cortico-cortical axons are myelinated in cerebral white matter, but they become less myelinated and thinner as they branch out in grey matter and ultimately form axon terminals. They also tend to bend as they leave the white matter before ramifying within the cortex (terminal arborization). Similar considerations apply for intracortical axon collaterals of the local, outward-projecting pyramidal axons (Aberra et al., 2018). Axonal bends, branch points, and terminals, as well as fiber diameter non-uniformities, constitute potential sites of low excitation threshold in local axons and long axon collaterals that arise from out-projecting pyramidal axons and make synaptic contacts with local intracortical circuits (Ghosh and Porter, 1988; Yamashita and Arikuni, 2001). Due to axonal arborization, the axonal branches and terminals of pyramidal cells point in various directions (Figs. 1 and 2). This has important implications. First, activation of pyramidal neurons can also be achieved with electric fields perpendicular to their main somato-dendritic axis (Figs. 1 and 2). For the same reason, interneurons that have a more isotropic arborization can be activated at low thresholds as well. Finally, simulations with full neuron morphologies indicate that the region of neuronal excitation extends all the way to the top of the crown (see Figs. 5 and 6 in Aberra et al., 2020).

#### Intracortical activation propagates in orthodromic and antidromic directions.

The action potentials initiated directly by the TMS pulse propagate orthodromically to cause synaptic transmission at all downstream connections, including both outward projections as well as local connections via the dense intracortical axon collaterals. Theoretical considerations suggest action potentials may fail to propagate through axonal branch points in an activity-dependent manner if there is a change in electrotonic load; however, orthodromic action potentials have been shown to propagate reliably throughout intracortical axon arbors for physiologically relevant firing rates (<200 Hz) in numerous experimental preparations and brain regions (Foust et al., 2010; Hamada et al., 2017; Radivojevic et al., 2017; Ritzau-Jost et al., 2021). Propagation failure at branch points in the antidromic direction due to impedance mismatch may be more likely, as axon diameters tend to taper in the distal direction; still, modeling and experimental evidence suggests that at low firing rates, antidromic conduction is also reliable. Activation of white matter tracts has been shown to cause antidromic action potentials that invade cortical cell bodies and generate complex cortical reverberations via the dense intracortical collaterals with deep brain stimulation of the hyperdirect axons from motor and premotor cortex to the subthalamic nucleus (Kumaravelu et al., 2018; Li et al., 2012; Li et al., 2007) and electrical stimulation of pyramidal tract axons (Ghosh and Porter, 1988; Kraskov et al., 2020). Modeling evidence suggests that branch points with high diameter ratios of parent to daughter branch diameters would be most susceptible to antidromic propagation failure (Grill et al., 2008), which was observed for the axon of the largest pyramidal tract neuron (Betz cell) modeled by Salvador et al. (20 μm main axon: 6 μm collateral) (Salvador et al., 2011). Therefore, the action potentials initiated by TMS are expected to propagate reliably throughout the cortical axon collaterals for stimulation frequencies relevant to TMS, but propagation failure may be possible in some morphologically unique cell types, such as the large L5 pyramidal cells in M1 (e.g. Betz cells). The initiation and propagation of action potentials in these axons may warrant further investigation. For example, it is unknown what role, if any, failure of antidromic propagation may play in the generation of oscillatory activity following single and repetitive TMS.

#### Myelinated axons of inhibitory interneurons also constitute targets for TMS.

As pointed out in section 2.1, TMS of M1 produces inhibitory effects at stimulus intensities that are subthreshold for evoking a motor response, suggesting that inhibitory interneurons might be an important target for TMS. An important factor that determines the susceptibility of intracortical interneurons to TMS is the relative degree of axonal myelination. In the somatosensory cortex of mice, half of the myelin in layer II/III and a quarter of the myelin in layer IV belong to axons of inhibitory interneurons (Micheva et al., 2016; Stedehouder and Kushner, 2017). These studies demonstrated that myelinated inhibitory axons predominantly belonged to parvalbumin-positive basket cells (Micheva et al., 2016; Stedehouder and Kushner, 2017). Biophysical modeling of TMS-induced excitation showed neural excitation of inhibitory basket cells at their axon terminals in the precentral crown at a wide range of stimulus intensities (Aberra et al., 2020). While there was substantial overlap in threshold distributions, the modeled basket cells displayed slightly higher thresholds for direct activation relative to the modeled pyramidal cells, particularly in L5, which can be attributed to their smaller axon diameters (Aberra et al., 2018). There are no measurements of the S-D time constants of intracortical inhibitory neurons, which could lend insight into their direct activation by TMS relative to the excitatory pyramidal cells.

At very low TMS intensities, inhibitory neurons may primarily be excited transsynaptically via low-threshold excitatory inputs. This notion is also supported by the results of a triple-pulse TMS study, in which two sub-threshold conditioning pulses increased the inhibition of the motor output evoked by a supra-threshold test stimulus (Bestmann et al., 2004). Although inhibitory neurons possess smaller cell bodies and are less myelinated than pyramidal cells, inhibitory interneurons appear to have a lower threshold for eliciting action potentials (Kawaguchi and Kubota, 1997). This can be, at least partially, attributed to the fact that interneurons have a higher input resistance than pyramidal cells (Pashut et al., 2014; Pashut et al., 2011; Radman et al., 2009). The higher input resistance facilitates somatic depolarization by synaptic currents, which may lower the threshold for interneurons to fire an action potential in response to indirect, synaptic activation (Pashut et al., 2014; Pashut et al., 2011; Radman et al., 2009). Another explanatory factor may be the physiological properties of their synaptic inputs. For instance, the fast-spiking, parvalbumin-positive interneurons may generate action potentials at higher probability and with shorter latency than other cells in response to TMS because they receive frequent and strong excitatory inputs (Beierlein et al., 2003; Povysheva et al., 2006). Thus, concurrent TMS-induced excitation of axon terminals projecting from pyramidal cells onto an inhibitory interneuron would elicit synchronous synaptic inputs and raise the likelihood of action potential initiation at low stimulus intensities, as suggested from human TMS studies (Berger et al., 2011; Ziemann et al., 1996d).

TMS may also directly activate the axons of inhibitory interneurons, which show considerable spatial dispersion and arborization in grey matter (Tanaka et al., 2011). The largely isotropic spatial dispersion of axon branches may explain why the inhibitory electrophysiological TMS effects that can be produced by stimulating motor cortex are relatively robust against changes in orientation of the induced tissue current (Ziemann et al., 1996d). It is also worth pointing out that the axons of different types of inhibitory interneurons differ considerably in their electrophysiological properties, which may lead to cell-type specific neural response profiles to TMS (Casale et al., 2015). Still, it remains unclear to what extent the acute inhibitory effects of TMS are mediated by direct or indirect activation of inhibitory interneurons, and it is possible that both mechanisms play a role.

#### An alternative account postulates that TMS primarily induces neuronal excitation at the axon hillock of pyramidal neurons (Tranchina and Nicholson, 1986).

Pyramidal cells have a bipolar structure formed by the dendritic tree, soma and axon, being oriented perpendicular to the cortical surface. Tranchina and Nicholson used analytical analysis of basic cable theory and predicted peak polarization at the soma by uniform E-field due to impedance mismatch between the axon and dendrite (Tranchina and Nicholson, 1986). Some modeling work using compartmental neuron models found the soma is depolarized by a TMS pulse enough to trigger an action potential at the axon’s initial segment at threshold intensities (Pashut et al., 2011; Seo et al., 2016). According to this modeling work, the electric field hyperpolarizes the dendritic tree and depolarizes the soma and axon, if an electric field is directed from the dendritic tree towards the soma and runs in parallel to the somatodendritic axes of pyramidal cells. However, these studies used simplified model neurons with a single, straight main axon without including axonal arborization or axon terminals. Additionally, the results of the compartmental modeling studies by Pashut et al. and Seo et al. may have been caused by artefactual current generation at the interface between soma and the axon initial segment interface due to implementation errors in the E-field to neuron coupling, discussed in detail in (Wang et al., 2018).

The phenomenological *cortical column cosine* theory also predicts strongest activation by the E-field orientation parallel to cortical columns, i.e. parallel to the main somato-dendritic axis of pyramidal (Arabkheradmand et al., 2018; Fox et al., 2004; Krieg et al., 2015). Due to this “axis sensitivity”, the normal E-field perpendicular to the cortical surface determines neural excitation. Like the compartmental modeling studies by Pashut et al. and Seo et al., the *cortical column cosine* theory postulates that depolarization takes place at the axonal hillock of the soma, referred to as “somal sensitivity” (Fox et al., 2004). Accordingly, the *cortical column cosine* theory predicts that the lip region of the precentral crown is the preferential target site for TMS because the lip region is the most superficial part of the gyrus where the cortical columns are optimally aligned to the normal electrical field produced by TMS (Fig. 2c) (Fox et al., 2004). In contrast, the top of the crown should not be susceptible to TMS, because the normal component of the electrical field is perpendicular to the orientation of the cortical column (Fig. 2b).

At variance with the notion of soma sensitivity, biophysical models that included realistic axon morphologies found that axon terminals have the lowest threshold for activation by TMS (Aberra et al., 2018; Wu et al., 2016). Based on these compartmental modeling studies, direct depolarization of soma or axonal initial segment is unlikely. Due to the weak E-field coupling at this point and high somatic capacitance, direct depolarization of the soma or axon hillock was less than ~ 2–3 mV at threshold. Hence, all the other axonal discontinuities (branch points, bends, and terminations) were well above threshold before the soma or axonal hillock were effectively depolarized. A preferential excitation of axonal discontinuities by the TMS-induced electrical field has important implications for the primary target site of TMS. Because electric fields perpendicular to the cortical column can effectively excite the axonal arbor of pyramidal neurons and interneurons, the crown of the gyrus constitutes a primary target site for TMS in addition to the lip region (Fig. 2b). For further discussion of the possible mechanisms and implications of somatic vs. axonal excitation, refer to section 3.4.

#### Attempts to estimate the coupling of the electric field to the neural target structure need to consider the individual cortical folding pattern.

Whatever the primary neural target of TMS may be, the sensitivity of such a target will strongly depend on its position in the induced electric field distribution, such as whether the cortical target is located more superficially at the crown or lip region of a gyrus or deeper in the wall or fundus of a sulcus. With respect to the local electric field, an axonal terminal, bend, or hillock in a cortical column can be exposed to substantially different electric field magnitudes due to the decay of field strength with increasing distance from the coil. At the same time, the spatial relationship between the axonal target structure and the electric field will change dramatically, altering the biophysical impact of TMS on the same type of neuronal target structure. For example, downward projecting axons of L5 pyramidal cells on opposite sides of the precentral gyrus have opposite orientations relative to the induced electric field, leading to depolarization of one population and hyperpolarization of the other for the same phase of the stimulus. Therefore, the exact determination of neuroanatomical “hot spots” in terms of TMS-induced neuronal activation at the microstructural level remains a challenge that can be addressed, in part, by coupled electric field and neuron models (Aberra et al., 2020) as well as imaging techniques, provided that they can resolve and are specific to direct activation of neural elements.

#### In summary, it is highly unlikely that TMS selectively excites exclusively a specific neuronal microstructure in the targeted cortex.

On the contrary, a multitude of neural target structures exist, e.g., terminals of myelinated axons of pyramidal cells or incoming projection neurons, myelinated axons of cortico-cortical or cortico-subcortical axonal projections, local myelinated axons of inhibitory interneurons, and (less likely) axonal hillocks of pyramidal cells. This heterogeneity in part explains the multitude of physiological effects that can be elicited with TMS (see section 2.1). Depending on its location with respect to the hemispherical surface, each target structure has a distinct spatial relationship with the TMS-induced electric field which determines the regional susceptibility of any neuronal target structure to inductive magnetic stimulation.

### 2.3. Insights from neuropharmacology

Pharmacological manipulations have been instrumental in three ways to enhance our knowledge about what is being stimulated by TMS. These different lines of research combined TMS with MEP recordings, EEG or positron emission tomography (PET), respectively.

#### The first line of research addressed the question of how drugs acting on the central nervous system can change the motor response that is evoked by TMS targeting M1.

Voltage-gated sodium channel (VGSC) blocker such as carbamazepine, lamotrigine or phenytoin increase the corticomotor threshold (Chen et al., 1997; Mavroudakis et al., 1994; Ziemann et al., 1996b). Since VGSCs regulate axon excitability, these findings support the notion from biophysical modeling and neurophysiological experimentats that the TMS-induced electric field primarily excites axons rather than the soma of neurons at the axonal hillock (Basser and Roth, 1991; Maccabee et al., 1993). Furthermore, positive modulators of synaptic inhibition acting on the GABA type A (GABA_A_) receptor, such as benzodiazepines and barbiturates, decrease MEP amplitude at stimulus intensities clearly above corticomotor threshold (Boroojerdi et al., 2001; Inghilleri et al., 1996; Schönle et al., 1989). This strongly suggests that TMS excites corticospinal neurons transsynaptically, and that activity of inhibitory interneurons controls this route of excitation (Amassian et al., 1987; Di Lazzaro and Ziemann, 2013). Benzodiazepines may also enhance the inhibition of MEP amplitude in short-interval paired-pulse TMS protocols (Ilic et al., 2002; Ziemann et al., 1996a, 1996b). This conditioning effect on MEP amplitude provided evidence for the notion that TMS activates GABA_A_-ergic interneurons synapsing onto corticospinal neurons (Kujirai et al., 1993), or onto the excitatory interneurons connecting to corticospinal neurons (Ilic et al., 2002). Finally, a variety of drugs acting as agonists or antagonists in the dopaminergic, noradrenergic, serotonergic and cholinergic neuromodulating neurotransmitter systems modify the magnitude of the motor evoked response as reflected by the mean MEP amplitude (for review Ziemann et al. (2015)). This body of data corroborates that single-pulse TMS excites corticospinal neurons indirectly through a transsynaptic route. In addition, it shows that activity of these neuromodulating neurotransmitter systems contributes to controlling this route of excitation (Di Lazzaro and Ziemann, 2013; Hasselmo, 1995).

#### A second line of pharmacological TMS studies utilizes EEG to record with scalp electrodes how drugs shape the TEPs (Ilmoniemi and Kicic, 2010).

Pharmacological TMS-EEG studies demonstrated that GABA_A_ receptor agonists enhance the N45 response evoked by single-pulse TMS of left M1 in the non-stimulated right hemisphere (Premoli et al., 2014), while an alpha-5 GABA_A_ receptor antagonist selectively decreases the N45 response without altering the local TEP at the site of stimulation (Darmani et al., 2016; Premoli et al., 2014). These findings corroborated the long-held notion that single-pulse TMS of human M1 activates distributed bi-hemispheric brain networks, including activation of GABA_A_-ergic inhibitory interneurons in the contralateral pericentral cortex (Bestmann et al., 2004; Ferbert et al., 1992). In addition to auditory co-stimulation, TMS causes considerable somatosensory co-stimulation of peripheral receptors (e.g., vibration) and myelinated axons (see section 2.1). Axonal excitation may occur in distal axon segments passing through the scalp region where the local TMS-induced electric fields are maximal or in the proximal foraminal segment of the facial and trigeminal nerves due to eddy currents in the cerebrospinal fluid. Pharmacological modulation of cortical somatosensory processing may contribute to drug-induced changes in TEPs and should be considered as an alternative mechanism (Conde et al., 2019).

#### Other studies combined TMS with PET to probe lasting effects of repetitive TMS (rTMS) of the neocortex on dopamine related neurotransmission in the human striatum without involving a pharmacological manipulation.

Using the dopamine receptor ligand [11C] raclopride, PET revealed a topographically specific increase of dopamine secretion in the ipsilateral caudate nucleus after rTMS had been applied to frontal neocortex (Strafella et al., 2001). This study was the first to use a patterned “alpha-burst” protocol, consisting of 10-pulse bursts at 10 Hz separated by an inter-burst interval of 10 s (Strafella et al., 2001). Compared to rTMS over a control site, participants showed reduced [11C] raclopride uptake in the dorsal caudate nucleus ipsilateral to rTMS of the dorsolateral prefrontal cortex (DLPFC) (Strafella et al., 2001), or in the putamen ipsilateral to rTMS of M1-HAND (Strafella et al., 2003). These findings are in good agreement with the known cortico-striatal connectivity of the DLPFC and M1-HAND, forming parallel segregated pathways (Draganski et al., 2008), and confirm that focal TMS can preferentially excite the cortico-striatal projections deriving from the cortical target area (Bestmann et al., 2004; Siebner et al., 2003). Using another dopamine receptor ligand [11C] FLB 457, PET revealed dopamine release in anterior cingulate cortex and the orbitofrontal cortex ipsilateral to 10 Hz rTMS of the DLPFC (Cho and Strafella, 2009), supporting the view of a significant influence of human frontal cortex on dopamine release in large-scale distributed cortico-cortical and cortico-subcortical networks. It should be noted though that these TMS-PET studies probed lasting effects of rTMS on endogenous dopamine release in the striatum. Therefore, they provide information about tonic changes in endogenous dopamine release after rTMS, but no insights into how a single TMS pulse or a short TMS train acutely affects striatal dopamine release.

These studies, combining TMS either with pharmacological manipulations or with pharmacological tracer imaging, have proven instrumental in understanding that TMS activates the human brain directly through axonal excitation as well as transsynaptically, and results in propagated activity in large-scale intra- and interhemispheric cortico-cortical and cortico-subcortical networks.

### 2.4. Insights from in vivo animal models and in vitro experiments

#### *In vivo* animal models in rodents and *in vitro* experiments on neuronal cultures have been used to elucidate the physiological responses elicited by TMS at a level of detail that cannot be achieved in studies on humans.

TMS studies in small animals and *in vitro* setups are best suited for demonstrating the direct effects of TMS on neuronal activity using either conventional electric or more sophisticated optical recordings of activity. The latter has the big advantage of not being confounded by an electrical stimulation artefact. Additionally, molecular and histological approaches can be used to follow stimulation-induced changes in transmitter release or gene- and protein-expression, which occur within minutes.

In terms of transferability to the situation in humans, a couple of limitations need to be considered: 1) When using small animals like rodents, the conventional TMS coils are relatively large with respect to the size of the brain, exciting relatively large tissue volumes compared to studies in humans, while also reducing the peak induced electric field strength due to the small head size (Alekseichuk et al., 2019). It very much depends on the type of coil, its positioning and the orientation of the induced electric field whether only parts of the brain or the whole brain and peripheral structures like the retina and face sensors and muscles are stimulated. Custom-made small coils (e.g., 8 mm) (Grehl et al., 2015; Zhang et al., 2017) allow more focal stimulation, but stimulation intensity is two orders of magnitude lower than required for suprathreshold stimulation. 2) It also needs to be borne in mind that there are substantial between-species differences in cortical and corticospinal macroscopic anatomy (e.g., gyrencephalic vs. lissencephalic neocortex). Rodents lack cortical gyrification, their cortex has a different cytoarchitecture, and they have no direct monosynaptic axonal projections from M1 to the motor neurons in the spinal cord. 3) Animal studies often use anesthetics because animals otherwise would not tolerate TMS. Depending on the kind of substance used, anesthesia may dampen neuronal excitability in general or affect primarily excitatory or inhibitory synaptic transmission, thereby possibly interfering with the TMS effects. Animals can be stepwise adapted to the TMS procedure, but the acute stress level often remains elusive. 4) Furthermore, standard physiological measures such as the motor threshold cannot be measured at all or with less precision in animals, in particular with reference to resting vs. active corticomotor threshold. 5) Finally, differences in the auditory stimulation produced by the TMS clicking sound may be important as well. In small animals, the typical lack of hearing protection, thinner skull, proximity of the cochlea to the coil, and different hearing thresholds and frequency limits may alter the brain responses to TMS compared to human studies. Thus, sham or other control TMS conditions are important not only in human studies, but also in animal experiments.

*In vitro* studies of magnetic stimulation of acute brain slices, organotypic cultures or cell cultures allow for a better controlled geometry of induced electric fields and the measurement of acute and discrete changes in cellular processes. However, the translational value of such studies is limited because of the different physiological conditions of *in vitro* preparations compared to the state of an awake human brain controlled by numerous modulatory systems. Despite of these inherent limitations, animal research is critical to resolve what is being stimulated with TMS at the single-cell and cell-circuit level.

*In vitro* and *in vivo* recordings have not only been used to study the biophysical response properties of single neurons to inductive magnetic stimulation (covered in section 2.2) but also to delineate which types of cortical neurons are activated by TMS and in which temporal order. By the aid of sophisticated custom-made recording systems, which suppress the stimulation artefact, it could be demonstrated that a single TMS pulse evokes a sequence of excitatory and inhibitory responses of neuronal activity with the shortest spike responses within 1–6 ms. By recording neural spiking activity in the neocortex of macaques, Mueller et al. (2014) showed that a single TMS pulse evoked a sequence of action potentials which can be interpreted as an initial response of an axon, followed by that of an inhibitory interneuron and finally by a pyramidal cell. In rat motor cortex, Li et al. (2017) demonstrated that the pattern of short-latency evoked spiking activity varies with the orientation of the induced electric field. While mediolateral (M−L) stimulation, even at high intensity (120% MT), scarcely evoked any spike, P-A stimulation evoked robust firing with peaks at 1.2–1.6 and 3.2–4.2 ms, reminiscent of the I-wave pattern in human M1. Both orientations of TMS produced MEPs, but lateral-to-medial (L-M) stimulation did not lead to early increases in cortical discharge. This pattern led the authors to conclude that L-M stimulation resulted in direct subcortical stimulation of corticospinal axons. Typically, this early response was followed by an excitatory volley peaking around 20 ms and thought to rely on recurrent activity via the basal ganglia loop and cerebellum, followed by an inhibitory phase of 100–200 ms, which is terminated by a rebound excitation (Li et al., 2017). This inhibitory phase is likely mediated via activation of GABA_B_ receptors (Murphy et al., 2016), see below). Similar sequences of excitatory and inhibitory volleys of activity evoked by a single TMS pulse have been reported for monkey motor cortex (Tischler et al., 2011) and cat visual cortex (Moliadze et al., 2003). A recent study performed single-cell recordings in two rhesus monkeys (Romero et al., 2019): since a stimulation artifact precluded any recordings during the first 10 ms after the TMS pulse, that study did not probe the early direct response to TMS within the first 10 ms after TMS. Nonetheless, recordings revealed a range of effects of TMS on single-cell spiking activity (Romero et al., 2019). The most frequent cellular response to single-pulse TMS was a burst detectable at 10 and lasting up to 40 ms after the TMS pulse. Moreover, the effects of TMS on spiking activity were highly focal, as they were restricted to a cortical area measuring less than 2 mm in diameter.

In addition to invasive electrophysiological measurements, a range of novel optical imaging technologies have become available that offer high functional specificity as well as spatial and temporal resolution while avoiding contamination by electromagnetic artifacts (Kozyrev et al., 2014). Optical imaging of acute changes in neuronal activity in cat visual cortex using voltage-sensitive dyes revealed that a single TMS pulse causes a brief period of focal activation followed by a suppression of neuronal activity that lasted several hundred milliseconds (Kozyrev et al., 2014). A five-pulse train of 10 Hz rTMS led to a cumulative increase in overall postsynaptic potential levels, indicating the induction of a gradually increasing excitatory state across large neuronal populations during and shortly after the rTMS train (Kozyrev et al., 2014). Results from another recent rodent *in vivo* TMS study with a (M-L) orientation of the induced electric field favored the activation of callosal axons and emphasized the involvement of inhibitory interneurons (Murphy et al., 2016). Two-photon calcium imaging of neurons in layers I, II/III and V revealed that a preceding TMS pulse inhibited sensory responses of layer V pyramidal cells via a di-synaptic pathway. The pathway involved glutamatergic input to layer I and II/III interneurons mediating GABA-ergic inhibition to the apical dendrites of the layer V pyramidal cells based on GABA_B_ receptor activation. On the other hand, neither a direct (somatic) excitation of neurons by the TMS pulse was evident, nor an activation of thalamocortical inputs.

#### *In vivo* studies in animals have also disclosed short-lasting neuronal after-effects following the administration of single TMS pulses or short TMS trains (bursts).

A seminal study by Allen et al. (2007) applied short TMS pulse trains to visual cortex of anesthetized cats and performed simultaneous measurements of tissue oxygen and neural activity. TMS trains gave rise to a marked increase in spontaneous neural activity, which was dose-dependent and lasted for about one minute. This increase in “resting” activity was paralleled by a prolonged suppression of evoked neural responses to visual stimulation for 5–10 minutes and reduced phase-locking of spiking activity to intrinsic theta oscillations (Allen et al., 2007; Pasley et al., 2009). The TMS-induced changes in neural activity were reliably reflected by the dynamic changes in tissue oxygenation – a finding, which is of relevance to functional neuroimaging of TMS effects in humans (Allen et al., 2007). Follow-up studies revealed substantial trial-to-trial variability of the TMS-induced neural responses and linked this variability to the physiological state of the cortex at the time of TMS (Pasley et al., 2009) and reported a TMS-induced narrowing of the width of orientation tuning curves, indicating altered visual processing (Kim et al., 2015).

#### *In vivo* studies in animals have also verified the emergence of cortical plasticity following the repeated administration of TMS pulses.

Using a TMS-setup suitable for inducing callosal activity with a mediolaterally oriented electric field, it has been shown that rTMS induces changes in neuronal activity markers primarily within cortical layers II/III (Benali et al., 2011; Mix et al., 2010). Using an intermittent theta-burst stimulation (iTBS) protocol (Huang et al., 2005), rTMS markedly reduced the expression of parvalbumin in the fast-spiking interneurons. This finding indicates reduced activity of this interneuron population resulting in cortical disinhibition (Benali et al., 2011). These after-effects on inhibitory interneurons are in good agreement with the acute single-pulse effect of TMS on inhibitory neurons targeting dendrites of pyramidal cells when an electric field of M−L orientation had been induced (Murphy et al., 2016). Changes in parvalbumin expression could be achieved with a stimulation intensity of between 23 and 30% of maximal stimulator output (MO), indicating that activation of the long callosal axons with an induced electric field oriented parallel to the axons requires much lower stimulation intensity as for activating rat corticospinal projection cells with P-A orientation of the induced electric field (~80% MO) (Rotenberg et al., 2010). Signs of reduced cortical inhibition after high-frequency rTMS (10 Hz) were also obtained with optical imaging of cat visual cortex activity (Kozyrev et al., 2014). The short-latency inhibition induced by a single TMS pulse was markedly reduced after 10 Hz rTMS (Kozyrev et al., 2014) and plasticity of the cortical orientation map could be induced during this phase (Kozyrev et al., 2018) concordant with the hypothesis of disinhibition as a circuit mechanism to enable neuronal plasticity (Letzkus et al., 2015).

#### Subthreshold effects of TMS also need to be considered.

The question of what is stimulated by TMS also includes the functional impact of TMS-induced electric fields that are subthreshold to elicit action potentials. In principle, any “hot spot” of suprathreshold TMS is surrounded by brain regions of subthreshold stimulation level, but even in a “hot spot” only a portion of neurons might be effectively discharged while the local electric field may remain subthreshold for other neurons. Subthreshold de- or hyperpolarization of the neuronal cell membrane can affect synaptic responses and the orthodromic and antidromic propagation of action potentials, even if not directly eliciting action potentials. Although primarily demonstrated with repetitive stimulation (extremely low-intensity magnetic fields, usually about 50 Hz), magnetic fields of 1 mT, or even less, are able to change intracellular calcium levels and the activity of downstream molecular pathways (Carlezon et al., 2005; Grehl et al., 2015; Zhang et al., 2017).

### 2.5. Insights from functional brain imaging

#### Functional neuroimaging has been used intensively to capture the acute functional impact of TMS on human brain networks.

Online neuroimaging can detect acute effects produced by TMS in any cortical and subcortical region throughout the brain and with high spatial and temporal resolution (Bergmann et al., 2016; Bergmann et al., 2021; Siebner et al., 2009). This is relevant because the neural response to TMS can otherwise only be quantified directly over M1 through MEP recordings or at sensory areas by quantifying psychophysical responses (e.g., Paulus et al. (1999b) or phosphenes (Kammer et al., 2005a; Kammer et al., 2005b).

#### TMS-neuroimaging studies have consistently shown that TMS can modulate ongoing neural activity in distributed brain networks.

PET and functional magnetic resonance imaging (fMRI) provide whole-brain coverage at good spatial resolution (Bergmann et al., 2016; Siebner et al., 2009). A critical difference between online TMS-PET and online TMS-fMRI regards their temporal resolution. While PET has a poor temporal resolution, ranging from tens of seconds to minutes depending on the radioligand, temporal resolution of fMRI is in the range of a few seconds. This implies that single PET scans of regional cerebral blood flow (rCBF) or regional metabolic rate of glucose (rCMRglu) average the evoked activity of long trains of rTMS that lasts at least tens of seconds (Fox et al., 1997; Paus et al., 1997, 1998; Siebner et al., 1998b). Hence, the resulting activation maps reflect an averaged read-out of TMS-induced regional brain activity, which might be influenced by acute neuromodulatory effects of TMS on brain activity that emerges during the rTMS train. In contrast, the better temporal resolution of TMS-fMRI enables the study of regional activation evoked by a single TMS pulse or a short TMS burst (Baudewig et al., 2001; Bestmann et al., 2003, 2004; Bohning et al., 1998).

Parametric PET studies of rCBF and rCMRglc showed that rTMS trains not only lead to dose-dependent activity changes at the cortical stimulation site, but also in remote cortical regions known to form a functional network (Fox et al., 2006; Laird et al., 2008; Paus et al., 1997, 1998; Siebner et al., 1998b). This work in human volunteers has been complemented by TMS-PET studies in the baboon (Salinas et al., 2016; Salinas et al., 2013). Similar results were observed by interleaving short high-frequency bursts of TMS with fMRI over different cortical sites including premotor and motor cortices (Baudewig et al., 2001; Bestmann et al., 2003, 2004; Bohning et al., 1998). Both local and network changes are generally dose-dependent and increase with increasing stimulation duration or intensity. For example, a short train of TMS over M1, even at intensities that are subthreshold for MEPs, may lead to remote activity changes in sensorimotor regions (Bestmann et al., 2003). Yet acute TMS effects may remain restricted to the targeted cortex, if TMS is applied at relatively low stimulus intensities (Siebner et al., 2001; Takano et al., 2004).

It is worth noting that a local increase in the BOLD response measured from structures situated directly under the stimulating coil is not always seen in TMS-fMRI studies (Bergmann et al., 2021). This may be attributed to suboptimal target engagement. For instance, stimulation intensity, number of pulses or coil positioning over the target site may have been insufficient to reliably engage the specific cortical region. The absence of a local increase in the BOLD signals may also reflect complex interactions between TMS-evoked neuronal activity and the associated shifts in the excitation/inhibition balance and the metabolic and thereby neurovascular response. Technical aspects, such as a relatively low signal-to-noise (SNR) ratio may also play a role. These factors, alone and in combination, may complicate providing proof of local target engagement in concurrent TMS-fMRI studies. For a detailed discussion of these aspects, we refer to Bergmann et al. (2021).

Perhaps the most relevant contribution of TMS-neuroimaging studies has been the confirmation of the idea that TMS to a cortical site can also influence activity in subcortical brain regions (Bestmann et al., 2004; Blankenburg et al., 2008; Strafella et al., 2001; Strafella et al., 2003), as well as cortico-subcortical connectivity (Herz et al., 2014). Another important and consistent finding was that the influence of focal TMS on regional brain activity is modulated by the state of the targeted brain network (Bestmann et al., 2008; Blankenburg et al., 2010; Moisa et al., 2012; Ruff et al., 2008) and that this influence is modulated by the state of both the targeted area and network (Bestmann et al., 2008; Moisa et al., 2012).

TMS may also be combined with EEG recordings. EEG captures the cortical response to single TMS pulses with high temporal resolution. Online TMS-EEG studies have consistently shown that a single TMS pulse gives rise to a rapid propagation of activity across putatively interconnected regions, including areas in the hemisphere contralateral to TMS (Ilmoniemi et al., 1997; Massimini et al., 2007; Massimini et al., 2005). The cortical target of TMS may thus serve as an entry point to non-invasively alter activity in specific subcortical structures and in cortico-subcortical networks, with predictable behavioral (Herz et al., 2014; van Schouwenburg et al., 2012) and potentially therapeutic consequences. Combined TMS-EEG studies further showed that regional cortical reactivity of the stimulated cortex differs in terms of the prevailing oscillatory activity evoked by TMS (Fecchio et al., 2017; Rosanova et al., 2009). Collectively, TMS-EEG studies have established that TMS can activate large brain networks. This activation can be very rapid and can occur even with single-pulse TMS at subthreshold intensities.

#### Combined TMS-neuroimaging studies can pinpoint brain activity changes that are elicited by sensory effects of TMS.

In addition to its transcranial mode of action, TMS excites the brain through afferent neuronal channels activated by concurrent auditory and somatosensory stimulation (Bestmann et al., 2004; Siebner et al., 1999). TMS causes peripheral stimulation of the central nervous system through multiple channels, including peripheral receptors and peripheral myelinated axons. The sources of peripheral co-activation are covered in section 2.1 and illustrated in Fig. 3. These peripherally induced effects may also contribute significantly to the temporo-spatial propagation of cortical activation seen after a single TMS pulse with EEG (Conde et al., 2019) or after short high-frequency TMS bursts with fMRI (Bestmann et al., 2004). While perhaps not surprising, visualizing the magnitude of these peripheral effects in terms of evoked brain activity may help to dissociate direct TMS-induced changes in brain activity from indirect activity changes related to sensory processing of TMS-induced inputs.

Due to its low temporal and spatial resolution, proton magnetic resonance spectroscopy (proton-MRS) has preferentially been used to capture regional metabolic changes after prolonged TMS (i.e., rTMS protocols) (Stagg, 2014). Yet, proton-MRS has also been used to gain insights into the metabolic underpinnings of TMS-derived metrics of cortical excitability (Harris et al., 2017). For instance, proton-MRS has been used to relate MEP-based measures of cortical excitability to regional levels of glutamate and GABA in the motor cortex (Stagg et al., 2011).

#### Online TMS-neuroimaging studies corroborate the notion of “state dependency” (Fig. 4).

**Fig. 4.**
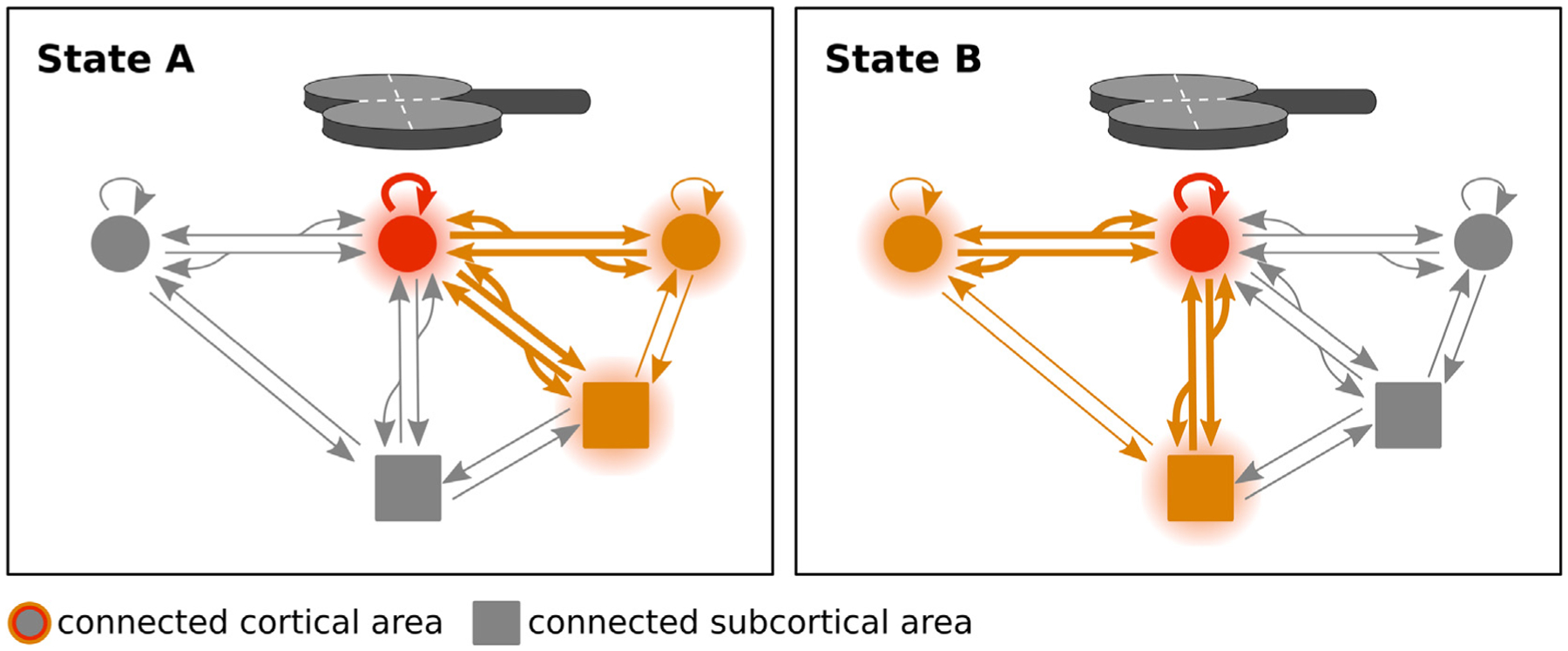
Network effects of transcranial magnetic stimulation (TMS) and state-dependency. Focal TMS can induce neural activity in nodes of the brain network connected with the targeted cortical region. Excitation of connected regions occurs through axonal and transsynaptic conduction of the regionally induced action potentials to anatomically connected cortical and subcortical regions. Axonal spread may also involve antidromic excitation. The propagation of neuronal excitation throughout the network depends on its physiological state at the time of stimulation. This is illustrated conceptually in the network diagram. Depending on whether TMS is applied in state A or state B, the network propagation that is evoked by a physically identical TMS pulse given over exactly the same cortical region (red) with the same intensity, may differ substantially not only in magnitude but also in spatial pattern. State dependence may be more relevant to orthodromic propagation as compared to antidromic propagation throughout the targeted brain network.

As pointed out at the end of section 2.1, the physiological consequences of TMS strongly depend on the functional state of the targeted cortical region at the time the TMS pulse is applied. For example, the size of local and remote activity changes in response to TMS may scale with the underlying state, for instance whether TMS over M1 is applied at rest or during voluntary movement (Bestmann et al., 2008; Bestmann et al., 2010; Paulus and Rothwell, 2016). It has been argued that the transmembrane resistance of a neuron is lower in an activated state, which renders any magnetic or electric stimulation less effective. The network changes elicited by TMS may not just depend on the state of the local targeted cortical site, but also on the activation state of putatively interconnected regions that form part of a functional network (Blankenburg et al., 2008; Blankenburg et al., 2010; Moisa et al., 2012; Ruff et al., 2008; Ruff et al., 2006). Together, the neuroimaging work has provided consistent evidence that TMS activates large brain networks, but differently so when being applied during different activation states of the targeted network components (Fig. 4) (Bortoletto et al., 2015).

### 2.6. Insights from dual-site TMS

#### Dual-site TMS provides a unique opportunity to test the causal neurophysiological interactions between interconnected brain areas.

Dual-site TMS paradigms generally combine the delivery of a conditioning stimulus over a cortical area to activate putative pathways projecting onto M1-HAND with a test stimulus over M1 (Koch, 2020; Koch and Rothwell, 2009). The test stimulus is suprathreshold to evoke a MEP. The size of the test MEP probes any changes in corticospinal excitability that are produced by the neural input to M1-HAND evoked by the conditioning pulse. Depending on the intensity and the inter-stimulus interval (ISI) of the conditioning stimulus both facilitation and inhibition may be detected in ipsilateral and/or contralateral M1 (Ferbert et al., 1992; Hanajima et al., 2001). Usually, the conditioning stimulus is applied before the test stimulus at ISIs that last up to tens of milliseconds. A notable exception is a dual-site TMS paradigm in which the test stimulus is given a few milliseconds before the conditioning stimulus to probe ultra-fast ipsilateral premotor-to-motor interactions (Groppa et al., 2012b). The dual-site TMS method has been widely used to study the physiology of cortico-cortical inputs to M1-HAND originating from interconnected areas such as contralateral M1-HAND (Ferbert et al., 1992), dorsal and ventral premotor cortex (PMd and PMv) (Baumer et al., 2006; Davare et al., 2009; Groppa et al., 2012b; Mochizuki et al., 2004), posterior parietal cortex (Koch et al., 2007), supplementary motor area (Arai et al., 2012) and somatosensory cortex (Brown et al., 2019). Dual-site TMS has also been used to study cerebellar-cortical interactions with the conditioning coil placed over the cerebellum and the test coil over contralateral M1-HAND (see section 4.6).

#### Most studies have attributed the conditioning effects of dual-site TMS to the excitation of a direct cortico-cortical pathway connecting the conditioned area and the stimulated M1.

This may, however, not always be the case. When considering that TMS of M1-HAND induces corticospinal descending volleys that leave the cortex several milliseconds after the TMS pulse has been applied, a conditioning pulse given several milliseconds before the test pulse has enough time to modulate the TMS effects in M1 via an indirect route that uses additional subcortical or cortical areas as relays. For instance, a polysynaptic pathway underlies cerebellar-cortical dual-site effects, because there is no monosynaptic connection between any cerebellar structure and M1-HAND. Tractography derived from diffusion weighted MRI scans may provide some hints on the white matter pathways that are engaged by a dual-site TMS paradigm. If microstructural properties of a certain white matter tract scales with the measures of effective connectivity, as obtained by dual-site TMS, this may increase the confidence that the physiological interactions are mediated through these cortico-cortical or cortico-subcortical tracts (Boorman et al., 2007; Fricke et al., 2019; Groppa et al., 2012c; Kloppel et al., 2008; Koch et al., 2011; Koch et al., 2010; Neubert et al., 2010; Wahl et al., 2007).

#### The functional interactions revealed by dual-site TMS are context dependent (Fig. 4).

The cortico-cortical interaction may vary when tested in the context of a task-free state (i.e., rest) or during a specific motor or cognitive task (Koch et al., 2006; Neige et al., 2021). These task-dependent changes give an indication of how the excitability of the connection changes over time when the cortical networks become active during a specific task (Groppa et al., 2012c; Koch and Rothwell, 2009). For instance, dual-site TMS revealed that effective connectivity between the posterior parietal cortex, premotor cortex, and M1-HAND increase in a highly task-, condition- and time-dependent manner during the planning phase of different reaching-to-grasp actions (Koch et al., 2010).

#### Deep brain stimulation (DBS) has recently been combined with TMS of M1-HAND to probe the conditioning effect of basal ganglia nuclei on M1-HAND.

The conditioning stimulus was applied through the implanted electrodes of the DBS device and triggered the TMS pulse (Ni et al., 2018; Udupa et al., 2016). In patients with advanced Parkinson’s disease who were treated with DBS of the subthalamic nucleus (STN), the implanted electrode was used to produce conditioning stimulation of the STN (Udupa et al., 2016). The conditioning STN pulse facilitated the MEP amplitude when given at a conditioning-test interval of 3–5 ms (early facilitation) and 18–25 ms (late facilitation) (Udupa et al., 2016). A subcortical-cortical conditioning-test approach was also applied in patients with dystonia treated with bilateral DBS of the internal globus pallidum (GPi) (Ni et al., 2018). A conditioning GPi pulse facilitated MEP amplitudes when the GPi pulse was given 10 ms before the cortical test stimulus (Ni et al., 2018). The same conditioning GPi pulse reduced MEP amplitudes when given around 25 ms before the test pulse to M1-HAND (Ni et al., 2018). Together, these DBS-TMS studies show that not only TMS-evoked inputs from the cortex and cerebellum, but also DBS-evoked inputs from the basal ganglia effectively shape the responsiveness of M1-HAND to TMS.

#### The timing-dependent, dual-site effects of TMS have been successfully used to induce spike-time dependent-like (Hebbian) plasticity with TMS.

Long-term potentiation (LTP)-like or long-term depression (LTD)-like effects in M1-HAND have been observed after dual-site paired associative stimulation (PAS) targeting M1-HAND bilaterally (Rizzo et al., 2009), PMv and M1-HAND (Buch et al., 2011), supplementary motor area and M1-HAND (Arai et al., 2011), posterior parietal cortex and M1-HAND (Koch et al., 2013), or ipsilateral basal ganglia nucleus and M1-HAND (Ni et al., 2018; Udupa et al., 2016).

#### Can the dual-site TMS approach be expanded to other cortical areas?

Conventional dual-site approaches have primarily targeted pathways that project onto M1, using the MEP evoked with a test TMS pulse over M1-HAND as a convenient read-out. In principle, dual-site TMS can also be applied to areas outside M1. This requires different physiological read-outs to probe the functional impact of the conditioning pulse. The combination of dual-site TMS with EEG has been proposed for this purpose using the TEP as read-out (Picazio et al., 2014; Veniero et al., 2013). Yet inherent methodological problems limit the use of EEG as a read-out for dual-site TMS. First, short-latency cortico-cortical interactions are difficult to trace due to the stimulation-induced artefact in the EEG. Second, it is problematic to infer directional causality from TEP recordings. The origin of the MEP can be ascribed to a certain cortical area, the precentral motor cortex, which greatly simplifies the interpretation of dual-site TMS studies in terms of directional causality. The situation is different, when recording TEPs which are complex network read-outs, reflecting the total activity across all areas. Therefore, both the conditioning and the test TMS pulse will evoke cortical potentials, which will result in complex reciprocal spatiotemporal interactions: The neuronal activity generated by the two TMS pulses may “arrive” at different times in different brain area, and this may happen recurrently within the stimulated networks. The cortical activity evoked by the first “conditioning” TMS pulse may modulate the TEPs evoked by the second “test” TMS pulse, but the cortical activity evoked by the second “test” TMS pulse may also modulate the TEPs evoked by the first “conditioning” TMS pulse. These reciprocal spatiotemporal interactions are most likely non-linear and cannot be disambiguated by simple subtraction. Hence, in contrast to conditioning-test paradigms based on MEP measurements, an unambiguous dissociation of “conditioning” and “test” effects is not possible for TEP-based dual-site read-outs. Thirdly, the conditioning and test TMS pulses cause paired somatosensory and auditory cortical responses (Conde et al., 2019). These peripherally evoked potentials will interact with each other and with the TEPs, complicating the interpretation of dual-site TEP experiments even further. The methodological concerns regarding dual-site TEPs also apply to conditioning-test TEP paradigms which examine paired-pulse TEPs evoked by single-site TMS targeting the same cortical area with a single transducing coil.

## 3. TMS targeting the primary motor cortex

**Fig. 5.**
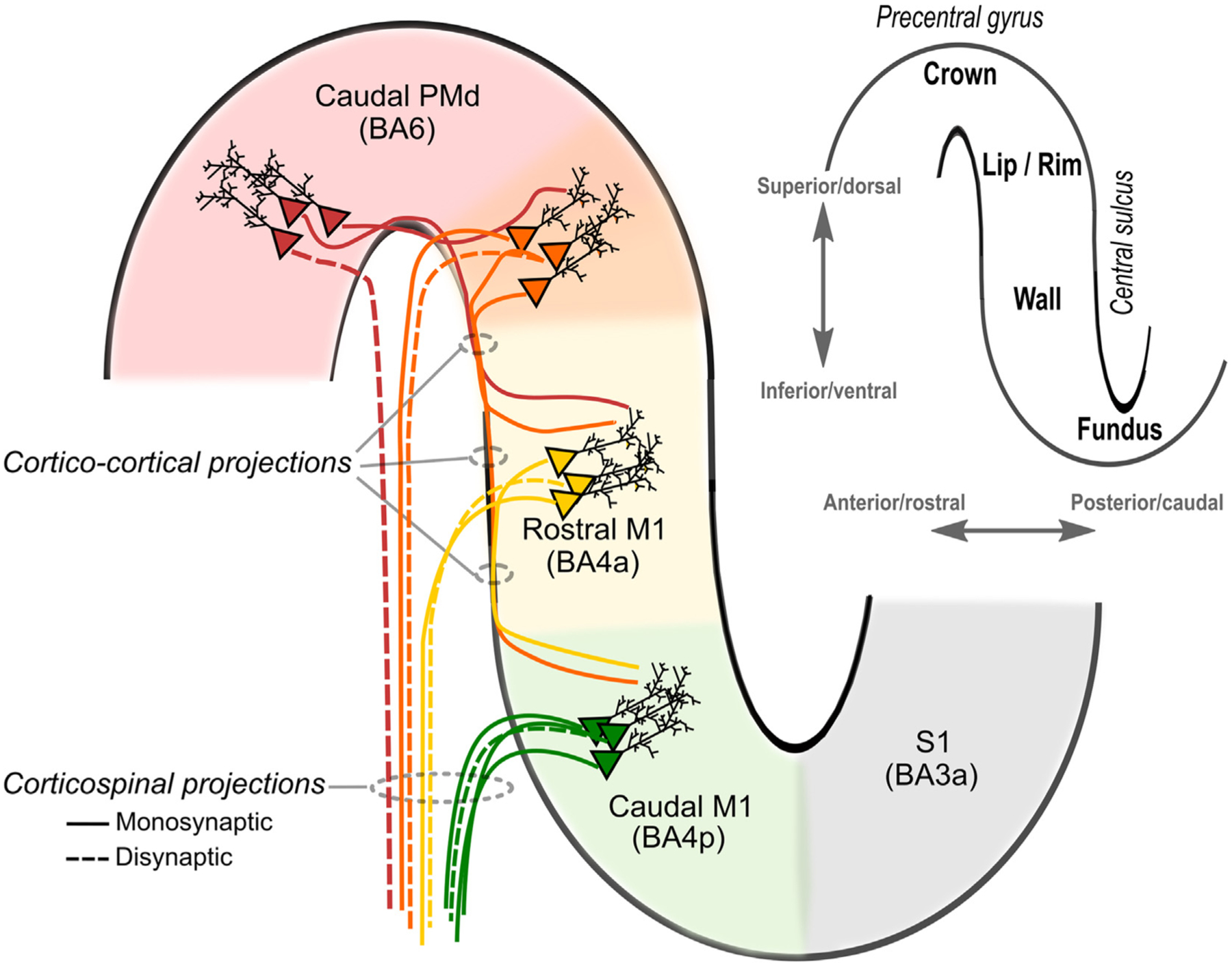
Candidate descending corticospinal pathways activated by transcranial magnetic stimulation (TMS) in the precentral motor hand knob. The insertion in the upper right-hand corner displays a sagittal slice of the motor hand knob with key anatomical landmarks highlighted. The likelihood of direct activation of neurons appears greatest in the lip/rim regions of the motor hand knob. Through synaptic transmission in cortico-cortical projections, activation will spread and activate rostral and caudal parts of M1 potentially contributing to indirect waves (I-waves). The greater preponderance of fast-conducting, monosynaptic cortico-motoneuronal neurons in the caudal (new) M1 (BA4p) compared to the rostral (old) M1 (BA4a) is highlighted. As shown, the exact transition between the rostral parts of the M1 and the caudal of PMd in the lip/rim region of the gyrus is gradual and may vary from subject to subject (highlighted in orange). Please note that this figure focuses on the precentral gyrus and anterior wall of the central sulcus, but additional corticospinal pathways may be activated by TMS via excitation of postcentral primary somatosensory cortex (S1) and its cortico-cortical projections to rostral/caudal M1.

The motor cortex rostral to the central sulcus, especially M1-HAND, has been the most popular cortical target for studies exploring the mechanisms of action of TMS (Fig. 1). Using the MEP of intrinsic hand muscles as functional read-out, these studies yielded fundamental insights into how TMS works. M1-HAND forms a characteristic knob-like structure which can be easily identified on structural MRI scans (Yousry et al., 1997). Due to its superficial location and its direct corticospinal projections to the cervical spinal motoneurons, the M1-HAND has been the preferential target site for TMS in the human M1 (Fig. 5).

When Barker and colleagues introduced TMS in 1985, they also targeted the M1-HAND. In their letter to Lancet, they included a figure featuring a MEP recorded from a hand muscle to illustrate the ability of TMS to probe the function of corticospinal projections (Barker et al., 1985). Indeed, the fact that one can elicit a motor response from a hand muscle by stimulating the contralateral M1-HAND provides clear evidence for an antegrade, transsynaptic propagation of neural excitation from the cortical target area to connected neural structures along pre-existing neural pathways. It is the transsynaptic propagation of excitation through which local TMS of a cortical area can produce remote excitation of interconnected cortical and subcortical areas, and motor neurons in the spinal cord. The short latency of the MEP indicates that the TMS-induced cortico-to-motor excitation propagates via the fastest-conducting large-axon corticospinal fibers which make monosynaptic connections with the cervical motor neurons.

Another important neurophysiological property of the MEP is that a slight pre-activation of the target muscle is sufficient to produce a consistent facilitation of the MEP compared to TMS with the target muscle at rest. This MEP facilitation is mediated by physiological changes at both spinal and cortical levels which renders the spread of local precentral excitation along the corticomotor pathways to the target muscle more efficient in a pre-activated relative to a resting state (Di Lazzaro et al., 1998b; Mills et al., 1987; Ugawa et al., 1995). This is probably the most compelling example of “state dependency” of TMS.

Although it is easy to record the MEP with surface electrodes, its underlying neurophysiology is complex. The MEP results from synchronized corticomotor excitation of fast conducting corticomotor neurons and propagation to the motor units of the target muscle (Siebner and Rothwell, 2003). Synchronization is not perfect and physiological properties of cell populations at the cortical, spinal and muscular level contribute to the MEP, resulting in substantial trial-to-trial variability of the MEP (for a detailed discussion see (Groppa et al., 2012a)). Phase cancellation causes a substantial decrease in MEP amplitude, even in healthy individuals, which can be largely eliminated by the triple stimulation technique (Magistris et al., 1998). Despite of the complexity of its underlying physiology, MEP-based neurophysiological studies have revealed important insights into how TMS acts on the M1.

### 3.1. Neurophysiological considerations

Some neurophysiological characteristics of TMS-evoked MEPs and their relevance in terms of the mechanism of action of TMS have shortly been mentioned in section 2.1 acknowledging their general relevance. In this section, we cover the neurophysiological characteristics of TMS applied to M1 in detail and relate them to the micro- and macroanatomy of the precentral gyrus.

#### 3.1.1. Transsynaptic induction of high-frequency volleys in corticospinal tract

When a slightly suprathreshold TMS pulse is given to the M1-HAND, multiple descending volleys can be recorded at short intervals from the corticospinal tract (Di Lazzaro and Rothwell, 2014) and multiple peaks of increased firing in the post-stimulus time histograms can be recorded from single motor units in contralateral hand muscles (Day et al., 1989). Hence, a single TMS pulse causes populations of fast-conducting neurons of the corticospinal tract to fire repetitively at very high frequency (~670 Hz). While the exact mechanisms that cause these multiple descending volleys in the corticospinal tract are still a topic of debate (see section 3.4 for a detailed discussion), their existence allow an important general conclusion about how TMS may work: It shows that a single TMS pulse can elicit a complex pattern of neuronal activity in the target network. The evoked activity patterns in the stimulated brain network are determined by the intrinsic neurophysiological and neuroanatomical properties of the stimulated cortex and interconnected brain regions.

Invasive recordings from electrodes implanted into the epidural space of the spinal cord also revealed that TMS evokes corticospinal descending volleys mainly via a transsynaptic mechanism (Di Lazzaro and Rothwell, 2014). At intensities slightly above corticomotor threshold, a monophasic TMS pulse, producing a P-A oriented current in the precentral gyrus, elicits only “indirect descending waves” (or “I-waves”). The term “I-wave” was coined by Patton and Amassian (1954) because these later responses, unlike the initial response (D-wave), did not survive cortical ablation. The term “I-wave” thus emphasizes an intracortical, presumably transsynaptic activation of fast-conducting corticospinal neurons in the M1.

TMS may also give rise to an earlier volley, the so-called direct wave (or “D-wave”) which is produced by direct activation of corticospinal axons in the subcortical white matter (Di Lazzaro and Rothwell, 2014). However, such direct axonal excitation of the corticospinal neurons occurs only at higher intensities of TMS and preferentially if TMS induces lateral-to-medial oriented currents in the precentral gyrus (Di Lazzaro and Rothwell, 2014). High-intensity, bi-polar or monopolar TES can also be used to evoke a MEP in contralateral hand muscles (Caramia et al., 1989; Merton and Morton, 1980). The corticomotor latency of MEPs is a few milliseconds shorter for TES than for monophasic P-A TMS (Caramia et al., 1989; Day et al., 1989; Sakai et al., 1997). This difference in corticomotor MEP latency between P-A TMS and TES further corroborates the notion that TMS targets primarily the axons of excitatory cortical interneurons that are up-stream to the corticospinal output neurons, producing action potentials in the corticospinal axon indirectly via transsynaptic excitation (Di Lazzaro et al., 2004; Mills et al., 1992; Sakai et al., 1997; Werhahn et al., 1994). The basic physiological mechanisms that underpin TMS-induced excitation of the corticospinal projections is covered in more detail in section 3.4.

In humans, the cortical circuits implicated in the generation of I-waves can be probed non-invasively with paired-pulse TMS targeting the motor hot spot in the precentral gyrus (Tokimura et al., 1996; Ziemann et al., 1998). A first suprathreshold pulse and a second subthreshold pulse, or two pulses with intensities just below MT are applied through the same coil at ISIs ranging from 0.5 to 5 ms. Paired-pulse TMS reveals distinct peaks of short-interval intracortical facilitation (SICF) at ISIs of approximately 1.4 and 2.8. and 4.2 ms, corresponding to the I-wave periodicity revealed by epidural recordings (Tokimura et al., 1996; Ziemann et al., 1998). SICF is commonly probed with monophasic pulse pairs targeting the M1-HAND, but can also be evoked with biphasic pulse pairs (Kallioniemi et al., 2018) and has also been observed when TMS targeted the leg representation (Chen and Garg, 2000). Therefore, it can be concluded that SICF reflects the strength of excitatory intracortical synaptic interactions and is widely expressed in the precentral cortex (Hanajima et al., 2002; Ziemann, 2020; Ziemann and Rothwell, 2000).

Biphasic TMS pairs, triplets, or quadruplets separated by an ISI that corresponds to the individual trough between the first and second SICF peak facilitates MEP amplitude across a wide range of TMS intensities (Kesselheim et al., 2022). Short-latency facilitation at trough latency was weaker than SICF at the first SICF peak, but the relative difference in facilitation decreased with increasing stimulus intensity. These findings indicate that biphasic multi-pulse TMS engages two mechanisms to produce short-latency corticomotor facilitation: An intracortical mechanism that is related to I-wave periodicity and engages fast-conducting direct projections to spinal motoneurons, and a second corticospinal mechanism that does not rely on I-wave rhythmicity and may be mediated by slower conducting indirect pyramidal tract projections from M1-HAND to spinal interneurons.

#### 3.1.2. Directional sensitivity of precentral neuronal populations to TMS

When using a figure-of-eight coil and a monophasic pulse configuration to stimulate the M1-HAND, MEP latency and amplitude depend on the current direction induced in the cortex (Day et al., 1989). When monophasic TMS produces a P-A current flow in M1-HAND with respect to the local gyral axis, the threshold for inducing a MEP is lowest. Higher stimulus intensities are required to evoke MEPs when the current flow in the precentral gyrus has an A-P direction with respect to the local gyral axis, and MEP latencies are 1–3 ms longer for monophasic TMS inducing A-P currents in M1-HAND as opposed to P-A stimulation. Mills et al. (1992) turned the orientation of a figure-of eight coil in steps of 45 degrees over the hot spot of M1-HAND and recorded MEPs in a contralateral hand muscle using eight different coil orientations (Di Lazzaro et al., 2004; Mills et al., 1992; Sakai et al., 1997; Werhahn et al., 1994). MEP amplitude differed substantially across the eight current directions. When keeping the stimulus intensity constant, the largest MEP responses were obtained when the induced current passed from posterolateral to anteromedial in M1-HAND, corresponding to a coil orientation of approximately 45 degrees with respect to the mid-sagittal line, or 90 degrees with respect to the local motor cortex axis (Di Lazzaro et al., 2004; Mills et al., 1992; Sakai et al., 1997; Werhahn et al., 1994). Together, these studies provided consistent evidence that a monophasic TMS pulse inducing a P-A current in M1−HAND will induce MEPs in the contralateral hand that have a lower threshold, shorter latencies, and higher amplitudes compared to a monophasic TMS pulse of equal stimulus strength that induces an A-P directed current.

The orientation-dependent differences in latency can be attributed to differences in preferential I-wave activation as revealed by epidural recordings of the descending corticospinal activity and single motor unit recordings (Di Lazzaro and Rothwell, 2014). The P-A oriented electrical current in the precentral crown evokes preferentially early I1-waves (i.e., monosynaptic inputs to corticospinal neurons), whereas the A-P oriented electrical current evokes preferentially later I-waves with longer corticospinal latencies (see also sections 2.1 and 3.4). Hence, A-P and P-A directed currents activate different neural populations which need less (P-A) or more (A-P) time to generate transsynaptic excitation of the fast-conducting corticospinal output neurons in M1-HAND. This directional specificity of monophasic TMS pulses is particularly prominent at relatively low stimulus intensities that are slightly above excitation threshold and tends to attenuate at higher intensities of stimulation, when many different neural elements are activated by TMS (Di Lazzaro and Rothwell, 2014).

Based on this work, it can be concluded that different neuronal populations or circuits are targeted in the precentral hand knob by TMS when using different coil orientations that produce differently oriented currents with respect to the local axis of the precentral gyrus (Halawa et al., 2019). This notion is further supported by paired-pulse TMS studies, showing that monophasic P-A and A-P stimulation produce differential effects on SICI as well as facilitation (Hanajima et al., 1998; Ziemann et al., 1996c). In general, the stronger the directional asymmetry of the TMS pulse, the stronger the difference in preferential stimulation of different neuronal populations (Sommer et al., 2006; Sommer et al., 2018). As discussed in section 2.1, biophysical modeling indicates that the site of preferential stimulation in the precentral crown-lip region can shift depending on the orientation of the induced current (Aberra et al., 2020). According to this model, a TMS pulse that induces an A-P oriented current in the precentral crown leads to an anterior spatial shift of the sites of neural activation relative to a TMS pulse that induces a P-A oriented current, which would lead to longer MEP latencies (Aberra et al., 2020). The neural populations activated by A-P and P-A currents also appear to play different roles in motor control. They have been reported to behave differently in response to some rTMS plasticity protocols in healthy individuals (Hamada et al., 2014; Tings et al., 2005). However, their relative sensitivity to directional TMS is compromised in patients with spinal cord injury (Jo and Perez, 2019).

It should be noted that biphasic (full-sine wave) pulses also display orientation dependent effects when targeting the precentral motor hand knob, though to a lesser extent than monophasic pulses (Aberra et al., 2020; Lang et al., 2006). This is explained by the longer second phase of the biphasic TMS pulse (Corthout et al., 2001); when the induced current during this phase is directed in the P-A direction in the precentral gyrus, MEPs have a lower MT compared to the stimulation with a biphasic pulse in the opposite direction (Kammer et al., 2001; Sommer et al., 2006; Weyh et al., 2005).

#### 3.1.3. Stimulation of cortical inhibitory interneurons

TMS of M1 also produces inhibitory effects that have a lower threshold than the one necessary to evoke a MEP (Classen and Benecke, 1995; Davey et al., 1994; Ziemann et al., 1996d). The TMS-induced excitation of GABA-ergic cortical interneurons is also discussed in section 2.1. Inhibitory effects of TMS are conventionally probed with paired-pulse protocols, such as SICI (Kujirai et al., 1993) or as a TMS-induced suppression of voluntary muscular activity, referred to as the cortical silent period (Inghilleri et al., 1993; Wilson et al., 1993). The inhibitory phenomena that can be probed with TMS of M1 are discussed in more detail in section 3.6 further below.

A modeling study using morphologically realistic models of inhibitory basket cells in lamina 4 of precentral cortex estimated that their axon terminals may have a relatively low threshold for TMS activation (Aberra et al., 2020). They also modeled lamina I neurogliaform cells, which exhibited substantially higher thresholds, suggesting minimal direct activation of these cell types at low stimulation intensities. Aberra et al. also conducted preliminary simulations of other inhibitory interneuron types, including large, nested, and small basket cells in laminae 2–6, and found these cell types had similar or higher thresholds to the lamina 4 large basket cells. However, relatively little is known about the specific axonal properties of different inhibitory interneuronal cell types, and existing work suggests they vary in their ion channel properties and excitability (Casale et al., 2015). Further experimental characterization and model development capturing the diversity of interneuronal axons is necessary to determine the thresholds for direct activation of inhibitory cortical neurons.

Alternatively, it may be that most interneurons are activated transsynaptically via excitation of axon terminals that belong to the axonal arbor of excitatory pyramidal neurons, which were predicted to have the lowest activation thresholds (Aberra et al., 2020). It would be useful to know more about the differential excitation of inhibitory interneurons since their axon terminals are distributed in different cortical layers, determining the spatial distribution of inhibition along the somato-dendritic axis of the pyramidal cells. Regardless of possible cell-type-specific differences in responsiveness to TMS, the important point is that the low threshold for activation of inhibitory interneurons means that TMS generally evokes a mixture of excitatory (glutamatergic) and inhibitory (GABA-ergic) effects in the targeted cortical area.

Although MEPs are sensitive to current direction, Ziemann et al (1996c) found no effect of current direction on SICI. They used two coils superimposed on each other so that they could maintain a P-A orientation of test pulse whilst rotating the direction of the conditioning pulse. Both the amount of SICI as well as its threshold were unaffected by conditioning coil rotation. The relative lack of orientation sensitivity would be consistent with the idea that the conditioning stimulus directly activated the axons of inhibitory interneurons, which show considerable spatial dispersion and arborization at the cell population level (Tanaka et al., 2011).

However, the situation may be more subtle (Di Lazzaro et al., 2017). For instance, an A-P oriented conditioning stimulus does not always suppress corticospinal volleys evoked by an A-P test pulse (Di Lazzaro et al., 2006). In addition, Hanajima et al (2008) found that SICI was reduced in patients with dystonia when evaluated with an A-P oriented conditioning stimulus but was normal using a P-A oriented pulse (Hanajima et al., 2008). Thus, it may be that inhibitory interneurons are also indirectly excited by TMS via a transsynaptic route, engaged by direction-specific excitation of axon terminals projecting on to interneurons. The relative weight of direct axonal excitation versus indirect transsynaptic excitation may differ across different types of intracortical interneurons and constitutes an important open question that should be addressed in future research.

### 3.2. Implications of anatomical features of precentral cortex

#### 3.2.1. Precentral motor hand knob

The part of the M1 hosting the motor representation of hand muscles (M1-HAND) has a convex shape with an outwards curvature towards the parietal cortex. As pointed out in the previous sections, the characteristic curvature of the central sulcus renders the M1-HAND easily recognizable as knob-like structure on the cortical surface or axial slices or of structural MRI scans. Therefore, Yousry et al. coined the term precentral motor hand knob (Yousry et al., 1997). TMS will most efficiently evoke a MEP in the contralateral hand if the TMS pulse induces a P-A current in the pre-central gyrus that is oriented perpendicular to the central sulcus (see section 3.1.2). Because of this direction sensitivity, the curvature of the M1-HAND needs to be considered when mapping the spatial corticomotor representation of hand muscles with TMS. In conventional TMS mapping studies, the coil orientation is kept constant across stimulation sites, ignoring the curved shape of the hand knob. This will therefore introduce considerable differences in the induced current direction in M1-HAND across stimulation sites, if the coil orientation is kept constant during the mapping procedure. A personalization of TMS mapping will be needed, if one wishes to ensure that the TMS-induced tissue current in the precentral hand knob always has the same (e.g., perpendicular) current orientation with respect to the surface of the precentral gyrus. Frameless neuronavigation enables site-specific adjustment of the coil position according to the local curvature of the precentral gyrus. Informed by the individual structural MRI, the coil orientation at each precentral stimulation site can be individually adjusted to the regional curvature of the hand knob, producinga current orientation that is always perpendicular to the sulcal wall (Raffin et al., 2015). This individualized sulcus-aligned mapping procedure has been successfully used to demonstrate a center-surround organization of short-latency afferent inhibition-facilitation in human M1-HAND (Dubbioso et al., 2017) and to trace use-dependent representational plasticity within the M1-HAND (Raffin and Siebner, 2018).

#### 3.2.2. The rostral and caudal part of M1-HAND

The human M1 is located in the anterior bank of the central sulcus, covering the caudal wall of the precentral gyrus (Geyer et al., 1996; Geyer et al., 2000). In non-human and human primates, the cytoarchitecture of M1 is characterized by a relatively low cell density, a poor lamination, the lack of granular cells in a functionally intact layer IV, and the presence of large pyramidal cells in area 4p (Betz giant cells) of layer V (Geyer et al., 2000). The fundus of the central sulcus marks the causal border of M1 relative to the primary somatosensory cortex (Fig. 5). While the posterior border of M1 is clearly demarcated, the anterior border of M1 is more gradual with the density of Betz pyramidal cells in layer V steadily declining along a posterior-to-anterior gradient (Geyer et al., 2000) (Fig. 5). Close to the parasagittal midline, the anterior border of the M1 reaches the crown of the precentral gyrus, whereas the anterior border recedes into the rostral bank of the central sulcus in more lateral parts of M1 on the hemispheric surface (Geyer et al., 1996). This implies that for the hand and face representations within M1, the superficial part of the precentral gyrus (i.e., the crown) which is closest to the TMS coil mostly belongs to the caudal part of the PMd with the M1-HAND extending to a variable degree into the posterior lip region of the precentral crown (Siebner, 2020).

The transition zone in which the rostral M1-HAND is gradually transformed into PMd may vary across healthy individuals and thus influence how the M1-HAND is stimulated by TMS (Fig. 5). This inter-individual variability of the transition between motor cortex and PMd in the crown of the precentral hand knob was disclosed in a recent study that employed biphasic TMS pulses and a two-dimensional sulcus-aligned TMS mapping procedure of the corticomotor representation of the contralateral intrinsic hand muscles (Dubbioso et al., 2021). Adjusting the target locations and induced current directions in the stimulated M1-HAND to the individual shape of the central gyrus, sulcus-aligned spatial TMS mapping revealed that the individual motor hotspot locations in the precentral gyrus varied along the rostro-caudal axis (Dubbioso et al., 2021). The more rostral the motor hotspot was located in the precentral crown, the longer was the corticomotor conduction time. “Hotspot rostrality” was more pronounced in individuals in whom MRI-based R1-mapping revealed a higher precentral myelin content. Together, these findings show a rostro-caudal spectrum of functional and structural properties in the precentral gyrus that are probably related to between-subject variations in the gradual rostro-caudal transition between M1-HAND and PMd in the precentral crown and link these variations to inter-individual differences in regional cortical myelin content as revealed by MRI-based R1-mapping (Dubbioso et al., 2021). These findings have important implications for functional TMS targeting of M1-HAND based on the individual motor hotspot. When applying TMS at individual motor hot spot location, one preferentially targets different motor and premotor neuronal substrates in the precentral crown in different persons, at least when using a biphasic pulse configuration. This hotspot related difference in anatomical targeting of the precentral gyrus may constitute a relevant source for inter-individual variability in the physiological responses of the corticomotor system to interventional TMS protocols.

In most TMS studies targeting the M1-HAND, the M1-HAND is considered as a single homogenous area. Work in non-human primates showed that the M1-HAND can be divided into a rostral (old) and caudal (new) part based on the absence (old) or presence (new) of cortico-motoneuronal cells (Rathelot and Strick, 2009). The rostral and caudal M1 form two parallel bands running in mediolateral direction along the anterior wall of the central sulcus (Rathelot and Strick, 2009). Retrograde anatomical tracing studies in rhesus monkeys revealed that only the caudal band of the M1 contains cortico-motoneuronal cells with descending axons that make direct synaptic contact with spinal motoneurons innervating shoulder, elbow and finger muscles (Rathelot and Strick, 2009). Accordingly, the caudal M1 has larger layer V pyramidal cells that make direct connections with the spinal motoneurons and has on average a lower threshold for eliciting movements with intracortical microstimulation (Stepniewska et al., 1993). Using intracortical electrical stimulation, a study in macaque monkeys confirmed that only the caudal (new) M1 contains pyramidal cells with *fast* monosynaptic corticospinal projections to the cervical spinal motoneurons (Witham et al., 2016). However, the study also showed that both, the rostral (old) and caudal (new) part of M1 host *slowly* conducting mono-synaptic corticospinal projections to the cervical motoneurons (Witham et al., 2016). Fig. 5 illustrates the potential implications of these neuroanatomical properties for TMS targeting of the precentral gyrus in the human brain, accounting for the fact thatthe human M1-HAND is not a homogenous area.

The homotopic representations of the rostral and caudal M1 are strongly and reciprocally interconnected (Stepniewska et al., 1993), but they differ with respect to their cortico-cortical and thalamo-cortical connectivity patterns (Holsapple et al., 1991; Matelli et al., 1989; Stepniewska et al., 1993, 1994). Regarding cortico-cortical connectivity, the caudal portion of M1 is connected primarily with somatosensory areas, while the rostral M1 is strongly connected with both premotor and somatosensory areas (Matelli et al., 1989; Stepniewska et al., 1993). These differences has led to the proposal that the rostral part of the M1 represents the phylogenetic “Old” M1 which has corticospinal neurons that “influence motoneurons indirectly through their connections with spinal interneurons”, while the “New” M1 is located caudally in the central sulcus and has corticospinal cells that make direct connections with spinal motoneurons responsible for highly-skilled movements (Rathelot and Strick, 2009).

There is convincing evidence that this rostro-caudal segregation also exists in the human M1-HAND and has been labeled Brodmann area BA4a and BA4p (Geyer et al., 1996; Geyer et al., 2000). The posterior (caudal) band of M1-HAND lies in the depth of the central sulcus, covering its anterior wall (Geyer et al., 1996; Geyer et al., 2000). The anterior (rostral) band is located more superficially in the sulcal wall with a smooth transition into the caudal PMd (Geyer et al., 1996; Geyer et al., 2000). As mentioned above, the transition from rostral BA4a to caudal PMd (BA6) varies from person to person and rostral BA4a may therefore extend into the posterior lip region of the precentral crown in some individuals (Fig. 5).

The rostro-caudal segregation of M1-HAND and its variable and smooth rostral border have important implications for the ability of TMS to stimulate M1-HAND. One may intuitively assume that focal TMS targeting the precentral gyrus causes a homogenous stimulation of the entire M1-HAND, but this is not the case. Since the strength of the induced electric field attenuates with the distance from the coil, the rostral M1-HAND (BA4a) in the upper wall of the precentral gyrus will always receive a stronger current than the caudal M1-HAND (BA4p) in the lower wall of the precentral gyrus. In other words, the rostral M1-HAND (BA4a) is more susceptible to TMS-induced neural excitation because it is closer to the stimulation coil. The relative magnitude of direct neural excitation of caudal versus rostral portions of M1-HAND will depend on the temporal properties of the stimulus, as well as properties of the different neuron populations.

This poses a problem: Although stimulation in rostral M1-HAND might activate corticospinal neurons, their slow conduction velocities would produce MEPs later than we observe. The shortest latency MEPs are evoked by a latero-medial (L-M) oriented TMS pulse or a single high-voltage transcranial electrical pulse (TES) (Edgley et al., 1992). The central conduction time of these MEPs (~5 ms) is so short as to be consistent only with transmission in rapidly conducting corticospinal axons with monosynaptic connections to spinal motoneurons. Monophasic TMS inducing a P-A oriented current in the precentral crown produces MEPs with onset about 1.5 ms later than the minimum (Di Lazzaro et al., 1998a). Since this involves at least one additional synapse in the cortex, the corticospinal conduction velocity must also be very rapid.

This raises the question of what is being stimulated. The MEP threshold for L-M oriented TMS is higher than for P–A, which may mean that the induced current can spread far enough into the anterior bank of the central sulcus to activate the large fast-conducting corticospinal neurons in area BA4p. The situation is not as clear for conventional P-A oriented TMS. One possibility is that despite its lower threshold compared with L-M oriented TMS, the induced current still manages to spread deep enough to activate synaptic contacts directly on the corticospinal neurons of area BA4p. This seems unlikely in view of all the evidence that activation occurs in the gyrus and tip of the anterior bank. A second possibility is that P-A TMS activates intracortical axons in the gyrus that monosynaptically excite fast-conducting corticospinal neurons in area BA4p (Yamashita and Arikuni, 2001). The additional conduction time plus synaptic connection might account for the additional 1.5 ms latency of P-A MEPs. Note that this possibility implies that some axons must have a low threshold for excitation that is compatible with that of synaptic terminals. One possible location would be at axonal bends in the just-subcortical white matter in the crown-lip region. Salvador et al. modeled both intra-cortical axon terminations and projection axons of pyramidal tract neurons with idealized morphologies, finding terminations were activated with the lowest stimulus intensities (64.8 – 65.7 A/μs), and the projection axons were activated at their axonal bends at higher intensities (90.9 – 105.9 A/μs) (Salvador et al., 2011). These relative thresholds are consistent with the recruitment order of early I-waves and the D-wave for monophasic, P-A TMS, suggesting I-waves are elicited by activation of intracortical axon terminals and the D-wave is elicited by activation of white-matter axon bends at higher stimulus intensities. Alternatively, it is possible that the D-wave is produced by activation of the intracortical collaterals of pyramidal tract neurons at their terminals, leading to antidromic propagation of the action potentials back to the main axon (Amassian et al., 1990).

The uncertainty about site of activation has one more implication. If TMS activates neural elements in the precentral crown, but recruits corticospinal neurons in the sulcal wall, where are I-waves generated? Although numerous models for I-wave generation exist (Ziemann, 2020), at present there is no information that can address this level of detail. However, since most I-waves are recruited at intensities significantly above active motor threshold, the question may not be relevant, since they could depend on activation of multiple neural elements spread over large volumes of tissue in areas BA4a, BA4p and even beyond (see below).

#### 3.2.3. The dorsal premotor cortex in the precentral crown

The superficial parts of the PMd located in the crown of the precentral gyrus represent a spatial hot spot for TMS-induced neurostimulation. This superficial part of PMd is closest to the coil and therefore is exposed to the strongest electric fields in the crown and lip regions of the precentral gyrus (see Fig. 1A and section 3.4). This part of the precentral gyrus is mainly covered by the caudal part of the PMd which belongs to Brodmann area 6 (BA6) (Fig. 5). Only the very rostral part of the rostral M1-HAND (BA4a) may extend into the posterior lip region (Geyer et al., 1996). Since the caudal PMd in the crown of the precentral gyrus is closer to the stimulation coil than the M1-HAND which is mostly buried in the anterior wall of the central sulcus, simulations predict a local maximum of the TMS-induced electric field in caudal PMd relative to the M1-HAND (Bungert et al., 2017; Laakso et al., 2014). Further, juxtacortical axons in the superficial white matter underlying the gyral crown are also likely candidates for stimulation with high enough stimulation intensities (Laakso et al., 2014; Opitz et al., 2011). It follows that axonal structures of M1-HAND in the sulcal wall cannot be directly excited by TMS without inducing a concurrent and stronger co-stimulation of the caudal part of PMd in the precentral crown-lip region, when targeting the precentral gyrus with TMS.

Research in non-human primates provided converging evidence for a strong functional interaction between PMd and M1, demonstrating dense reciprocal monosynaptic cortico-cortical connections between the two areas (Dum and Strick, 2005; Muakkassa and Strick, 1979). Intracellular recordings revealed short-latency excitation of intracortical neurons in layer III and V of M1-HAND 1.1–6.5 ms after electrical microstimulation of cortico-cortical projections originating from PMd (Ghosh and Porter, 1988). Most relevant to the question of how much concurrent TMS of PMd may contribute to TMS-induced excitation of fast-conducting corticospinal output neurons in M1 is the work by Amassian and colleagues published in 1987 (Amassian et al., 1987): focal electrical stimulation of the premotor or postcentral cortical surface yielded “very large periodic waves” in the pyramidal tract which were “often incrementing in amplitude until rapidly extinguishing” (page 85 in Amassian et al., 1987). After removal of precentral cortex, stimulation of postcentral surface was no longer able to induce descending waves in the pyramidal tract (page 86, Fig. 19 in Amassian et al., 1987). The authors argued that the abolished response proves “that they are mediated by transsynaptic activation of motor cortical pyramidal tract neurons” (page 85 in Amassian et al., 1987). It can be concluded that electrical stimulation of premotor and postcentral cortex can readily evoke multiple descending volleys (corresponding to I-waves) through excitation of cortico–cortical connections, which trigger the intrinsic generation of I-waves in M1 (Patton and Amassian, 1954). The authors speculated that surface stimulation of premotor or postcentral cortical sites may “generate both direct and indirect orthodromic discharges in corticocortical axons projecting to the motor cortex” (page 85 in Amassian et al., 1987).

These biophysical and neuroanatomical considerations strongly support the idea that concurrent excitation of neuronal elements in PMd (and possibly also postcentral somatosensory cortex) results in relevant transsynaptic excitation of neural elements in M1-HAND via short-range cortico–cortical premotor–motor connections (Yamashita and Arikuni, 2001). This indirect excitation of M1-HAND may thus contribute to the neurophysiological features that characterize the MEP. Cortico–cortical axons originating from pyramidal cells in rostral M1 (BA4a) or caudal part of the PMd (Yamashita and Arikuni, 2001) as well as from the postcentral somatosensory cortex forming the crown-lip region of the postcentral gyrus are candidate routes of this transsynaptic indirect excitation (DeFelipe et al., 1986).

As pointed out previously, TMS induces stronger electric fields in the caudal PMd, located in the precentral crown-lip region, than in M1-HAND, located mostly in the sulcal wall. This implies that intracortical inhibitory interneurons in PMd might be excited more strongly than their counterparts in M1-HAND located in the central sulcus (Figs. 1 and 5). Due to their cellular geometry, TMS-induced excitation of inhibitory neurons might be less dependent on the geometric relationship between the neuron and the induced current. Therefore, TMS targeting the M1-HAND should inherently produce a stronger intracortical inhibition in the PMd (located in the gyral crown) than in M1-HAND (located in the sulcal wall). It is conceivable that intracortical inhibition evoked in PMd contributes to the inhibitory effects that can be observed with paired-pulse TMS on MEP amplitude. For instance, a weak conditioning pulse may induce intracortical inhibition in PMd that weakens the efficacy of a subsequent stronger test response to efficiently excite cortico-cortical facilitatory input from PMd to M1.

In summary, the anatomy of the precentral hand knob has several important implications for TMS. First, the curvature of the hand knob needs to be considered when mapping corticomotor representations of M1-HAND or examining effects of direction specificity regarding the induced electric field in the precentral gyrus. Second, the precentral gyrus is not a homogenous area which is equidistant to the TMS coil, but it hosts the caudal PMd (BA6) in the crown-lip region and the rostral and caudal M1-HAND (BA4a/p) in the sulcal part of the gyrus. These regions differ in their sensitivity to be excited by TMS because of differences in coil-cortex distance and the spatial orientation of cortical axonal structures and the induced electric field. This implies that PMd and M1-HAND are concurrently stimulated by TMS, but also in an inherently different fashion. Importantly, the strongest “local dose” in terms of the induced electric field is achieved in the crown-lip region covered mainly by the caudal PMd and to a variable degree by the rostral M1-HAND (BA4a, old M1). However, direct activation of M1-HAND (BA4p, new M1) in the anterior wall may partly contribute to MEP generation when using higher intensities of TMS or when inducing a L-M directed current.

### 3.3. Insights from experiments and models of I-wave physiology

Experiments examining descending I-waves in the corticospinal tract have revealed important insights into how TMS activates the corticomotor system. Although other sections do refer to I-waves, we included a dedicated section to provide comprehensive coverage of this important topic. A single TMS pulse given to the human precentral motor cortex produces repetitive descending volleys in the fast-conducting axons of the corticospinal tract. In animals, several descending corticospinal volleys have also been identified after M1 stimulation: a short latency “direct” volley that is believed to originate from the direct activation of corticospinal axons (i.e., D-wave), followed by a series of later “indirect” volleys (i.e., I-waves) numbered according to their temporal order (Amassian et al., 1987). These multiple descending volleys are thought to be caused by a synchronized discharge of distinct intra-cortical circuits at differently grouped timings and possibly also repetitive discharges in single pyramidal tract neurons. Repetitive transsynaptic excitation through repetitive excitatory postsynaptic potentials from the interneurons may produce high-frequency repetitive firing in Betz pyramidal neurons because of their very short refractory period (Kernell and Chien-Ping, 1967).

In humans, the short-latency D-wave and the transsynaptically generated corticospinal I-waves have been recorded directly in conscious subjects who have had electrodes implanted surgically in the epidural space of the cervical cord for control of pain (Di Lazzaro and Rothwell, 2014). These lines of research in animals and humans have also revealed several physiological differences between the first (I1) and later I-waves: In monkeys, cortical cooling has a selective effect on late I-waves with no change in the I1-wave (Amassian et al., 1987). Similarly, in humans only late I-waves are suppressed by several paired-pulse stimulation protocols (Hanajima et al., 1998) and only the late I-waves are affected by pharmacologically induced changes in the level of on-going cortical GABA-ergic activity (Di Lazzaro and Rothwell, 2014). The differential effect of cooling on early and late I-waves led Amassian et al (1987) to speculate that multiple cortical elements can be activated by cortical stimulation evoking the different I-wave components. This may involve different neural elements, including both intra-cortical and cortico-cortical neurons (Amassian et al 1987).

The possibility that different cortical circuits could be activated using transcranial stimulation in humans was first suggested by the pattern of discharge of single motor units in the muscle evoked by TMS pulses over the motor cortex. A single pulse produced several peaks of increased firing probability that were presumed to result from arrival of excitatory postsynaptic potentials at spinal motoneurons from the D- and I-wave volleys. The data showed that later peaks could be evoked in isolation by changing the direction of the current induced in the brain (Day et al., 1989). The possibility of evoking later peaks of single motor unit activity in isolation became even clearer when a focal (figure-of-eight) coil capable of inducing more directed current was used (Sakai et al., 1997). Using a monophasic TMS pulse configuration, it was shown that a P-A directed electrical current perpendicular to the central sulcus usually evoked I1- and then later I-waves, whereas an A-P directed current only induced later I-wave activity. In addition, short-latency afferent inhibition (SAI) produced much greater inhibition of the I3-wave evoked by a P-A directed current than the I3-wave evoked by an A-P directed current, suggesting that the late I waves from P-A and A-P directions are mediated by different circuits (Ni et al., 2011a).

The contribution of different cortical circuits in I-wave generation is also supported by Maier et al. (2013) who recorded responses evoked by intracortical stimulation in monkeys both from the surface of the cord and from individual axons of corticospinal neurons at mid cervical level. As in previous studies, they recorded the D- and I-waves from the surface of the spinal cord, but they also made some new relevant observations while recording from individual axons. Together with the high frequency I-waves discharging at 600 Hz recorded from most of the axons, they found that some of the axons showed delayed discharges at lower frequencies and also recorded some temporally dispersed activity outside the main frequency peaks identifiable from surface recording. They proposed that this additional activity might be produced by corticospinal axons with slower discharge and conduction velocity. This conclusion is in good agreement with findings yielded by intracortical stimulation of cortico-motoneuronal connections in anesthetized macaques (Witham et al., 2016). In that study, a considerable portion of the cortico-motoneuronal connections from caudal (new) M1 that supply the forelimb generated short-latency monosynaptic potentials in cervical motoneurons (Witham et al., 2016). Stimulation of rostral (old) M1 also produced long-latency monosynaptic effects, but they were relatively weak compared to the effects evoked by stimulation of caudal (new) M1 (Witham et al., 2016). Together, these findings raise the possibility that the volley recorded from the surface is dominated by the fastest conducting axons whose activity may hide responses transmitted by slower-discharging axons. Invasive recordings in humans of the corticospinal activity evoked by different directions of the induced current in the brain by TMS have revealed similar findings in that the late activity evoked by A-P current appears to be less synchronized and, in some cases, of lower frequency and thus, may not, as previously thought, be represented by later I-waves (Di Lazzaro and Rothwell, 2014; Di Lazzaro and Ziemann, 2013). Indeed, while the more commonly recorded descending activity recorded both in animals and in humans is represented by very high frequency (approx. 670 Hz) I-waves with a fixed order of recruitment, by reversing the direction of the induced current in the brain it is possible to record descending activities with different frequencies. Occasionally, a descending activity with a frequency that is a subharmonic (333 Hz) of that of the high frequency I-waves, has been seen (Di Lazzaro et al., 2017; Di Lazzaro and Ziemann, 2013).

These observations indicate that the physiology of I-waves is complex. Several theories about I-wave production have been proposed. Initial theories postulated the existence of a chain of intra-cortical interneurons projecting upon the corticospinal cells and hypotesized that an intensity dependent activation of the different interneuron circuits produces early and late I-waves. More recently, it has been hypothesized that the I-waves might be produced by cortical networks with specific oscillatory properties activated by transcranial stimulation. Based on a canonical cortical circuit model, Di Lazzaro and Rothwell (2014) proposed that I-waves might be produced by the activation of excitatory bursting pyramidal cells with their soma located in cortical layers II and III with axons that project upon corticospinal cells. In this case, the I1-wave would be produced by monosynaptic activation and late I-waves by reverberating activity in the oscillatory circuit composed of layer II and III excitatory neurons and inhibitory interneurons (see also Esser et al. (2005); Seo et al. (2016)).

Other models are based on physiological properties of the pyramidal cells. Rusu et al. (2014), updated in Seo et al. (2016), proposed that early and late I-waves are produced by intrinsic membrane properties of corticospinal cells in response to a single input from layer II and III interneurons impinging onto different parts of the dendritic tree. The dispersion of the inputs along the dendritic tree, in particular to distal and basal dendrites, together with the spiking properties of corticospinal cells, is suggested to be responsible for I-wave generation. This concept might find some indirect support in the concept of “leaky membranes” under activation (Paulus and Rothwell, 2016) since late I-waves disappeared during motor preactivation (Ziemann et al., 1998). Back-propagation activated calcium spike firing is another physiological mechanism that may account for TMS-induced burst-like firing of fast-conducting neurons in the corticospinal tract (Larkum, 2013; Larkum et al., 1999, 2001; Ugawa et al., 2020). When a single back-propagating action potential coincides with a subthreshold distal excitatory postsynaptic potential, a burst of action potentials can be evoked in pyramidal cells (Larkum, 2013; Larkum et al., 1999, 2001; Ugawa et al., 2020). It has been proposed that this cellular property of pyramidal cells may account for TMS-induced high frequency oscillations of single corticospinal pyramidal cells without involving any cellular clustering (Larkum, 2013; Larkum et al., 1999, 2001; Ugawa et al., 2020). This multi-compartment model assumes that a single TMS pulse triggers an action potential in the proximal portion of corticospinal pyramidal axons in layer V. This may trigger a recurrent synaptic excitation through axon collaterals, generating a transsynaptic feedback input to the apical integration zone (i.e. the distal dendritic tree close to the cortical surface) at a certain delay. However, this model that only relies on cellular properties of the pyramidal cell remains speculative and does not account for many published findings: Patch-clamp recordings in layer V pyramidal cells of rat M1 (Larkum et al. 1999, 2001) never demonstrated an interval of 1.5 ms or less between the first two or any later action potentials to represent the interval between I1- and I2-waves or later I-waves in epidural spinal cord or SICF recordings, but rather intervals in the order of 5 ms or more (Larkum et al., 1999, 2001; Short et al., 2017).

Very fast oscillations with a frequency comparable to the I-waves (~600 Hz) have been observed in neocortex of rats and cats (Jones and Barth, 2002; Jones et al., 2000; Kandel and Buzsaki, 1997) and humans (Gobbele et al., 1998). These oscillations reflect neural activity at the cell population level, while single cells may fire at lower rates (but see evidence for burst firing patterns below). Therefore, it has been proposed that high frequency network synchrony could be produced by modes of synchrony termed “clustering” in which the network breaks into several clusters of neurons each of which discharges at single cell frequency and which results in a network frequency that is correlated with the number of clusters (Brunel and Wang, 2003). It is possible that strong TMS excitation could result in poly-synchronization of clusters of strongly interconnected excitatory and inhibitory cortical neurons that fire with millisecond precision producing the I-wave activity (Di Lazzaro et al., 2012). Interestingly, the delay between I-waves is 1.5 ms and with this delay, computational models of networks of highly connected excitatory and inhibitory neurons predict a peak of activity of 667 Hz (Brunel, 2000) corresponding exactly to the I-wave frequency. Because both high-and lower-frequency I-waves can be recorded after TMS, it is conceivable, that, depending on the characteristics of the TMS pulse, more than one oscillatory network can be activated providing several sources of inputs to corticospinal cells.

Electrophysiological recordings of descending corticospinal volleys probe the orthodromic conduction of action potentials along large fast-conducting axons. Small slow-conducting axons are heavily underrepresented when measuring corticospinal orthodromic and antidromic conduction, although they outnumber by far the larger fast-conducting axons (Firmin et al., 2014). This includes two classes of corticospinal axons with lower conduction velocity, monosynaptic axons making directconnections with cervical motoneurons and polysynaptic slow-conducting axons projecting onto spinal interneurons (Witham et al., 2016). Therefore, the electrophysiological studies on I-wave physiology tell little about the bulk of corticospinal neurons with smaller axon diameter and slower conduction velocities. The possibility that there is a large population of corticospinal fibers not explored by TMS is supported by electrophysiological findings in patients with hereditary spastic paraplegia who may show severe pyramidal signs associated with normal MEPs (Di Lazzaro et al., 1999). To explain this discrepancy, it has been suggested that the clinical evidence of corticospinal tract involvement in the presence of normal MEPs might be explained by selective involvement of a subpopulation of neurons, that can well be the small pyramidal tract axons, with a relative sparing of the large fast conducting corticospinal fibers. The latter are those consistently activated by TMS.

### 3.4. Insights from calculating the electric field induced by TMS

Field calculations and measurements help to understand how coil geometry, its position and the head anatomy affect the induced electric field evoked in the precentral motor cortex. Even simplified head models, such as spherical models (Eaton, 1992; Heller and van Hulsteyn, 1992; Roth et al., 1990), already give important insights (see section 2.2 for more detailed discussion): The electric field strength decays rapidly with distance from the coil, excluding the direct stimulation of subcortical areas (Deng et al., 2013; Epstein et al., 1990; Thielscher and Kammer, 2004). In addition, radial components of the electric field are also suppressed by the sphere-air boundary, independent of coil position and orientation. In practice, this causes the field direction to be approximately parallel to the inner skull boundary. It is also well established that figure-8 coils induce the strongest fields at positions close to the coil center (Ravazzani et al., 1996; Roth et al., 1990; Thielscher and Kammer, 2004). Studies employing more realistic head models and numerical methods such as finite element method (FEM) or boundary element method (BEM) (Salinas et al., 2009) generally confirm these findings (Bungert et al., 2017; Laakso et al., 2014; Opitz et al., 2011; Thielscher et al., 2011), but additionally show that the folding of cortical gyri and the conductivity anisotropy of white matter also affect the field: Higher electric field strengths are observed in the crowns of cortical gyri when the field is perpendicular to the gyral crest. In this case, the comparatively strong currents flowing in well-conducting cerebrospinal fluid enter the gyral crown rather than being shunted. This results in a local peak of the induced electric field in the cortex in the crown-lip region of the precentral gyrus. This is in line with physiological experiments showing that the optimal current orientation for precentral motor cortex stimulation, as determined by the corticomotor threshold, is perpendicular to the central sulcus (Di Lazzaro et al., 2004; Mills et al., 1992; Sakai et al., 1997; Werhahn et al., 1994). Of note, the local field orientation relative to the pial surface varies from oblique (i.e. neither fully normal nor tangential) at the position of the gyral lips to mostly tangential directly at the crown.

Regional spots of high electric field strength may also occur in the white matter underlying the crown of the gyrus (see Fig. 1A). This gradient can be explained by a jump in electrical conductivity at the interface between grey and white matter as well as anisotropy in the conductivity produced by alignment of downward-projecting fiber bundles, which are mainly perpendicular to the electric field direction (Opitz et al., 2011; Thielscher et al., 2011). It has been proposed that the transition zone between cortical grey and white matter may be a site for triggering an action potential, when using FEM models with a sharp conductivity border at the grey-white matter interface (Miranda et al., 2007). In reality, this conductivity transition is not as sharp, because the increase in myelination is more gradual when transitioning from grey matter to white matter (Zilles, 1990). However, even when using a volume conductor model that included this unnatural, sharp conductivity transition, a recent modeling study did not find activation in L5/6 pyramidal axons that crossed the grey–white matter boundary in the precentral gyrus (Aberra et al., 2020). Therefore, it is unlikely that excitation of myelinated axons at the transition from grey to white matter plays a relevant role at slightly suprathreshold stimulation intensities.

Instead, corticofugal axons may be activated in the white matter at their bends by electric fields directed outward at the bend. Given that the main axons projecting from the tip of the crown into the white matter are mostly perpendicular to the field, which is tangential to the scalp, it is unlikely that these axons are stimulated by this mechanism. However, axons projecting from the transition zone between crown and wall of the precentral gyrus will bend downwards into the white matter at angles approaching 90°. These axonal bends might constitute a low threshold site of excitation (Gomez-Tames et al., 2020; Salvador et al., 2011; Yamashita and Arikuni, 2001).

While the field calculations proved useful to generate hypotheses on the likely stimulation positions in the brain, more validation studies are still required. For instance, the validity of field calculations could be demonstrated by showing that they can predict electrophysiological properties of TMS. The issue of validation concerns both the predicted field pattern (as it might be imprecise due to model uncertainties) and the sometimes implicitly used assumption that mostly positions of high field strength are stimulated. Indirect support comes from a range of motor mapping studies which used TMS with figure-8 coils at the optimal orientation and consistently demonstrated that the center of gravity is situated above the crown of the precentral gyrus (Herwig et al., 2002; Inuggi et al., 2010; Niyazov et al., 2005; Sparing et al., 2008), in line with the results of the studies modeling the TMS-induced electric field (Diekhoff et al., 2011; Dubbioso et al., 2021; Weiss et al., 2013). In addition, in patients, a good overlap between a “mean stimulation field” reconstructed from TMS motor mapping via FEM calculations and direct electrical stimulation was demonstrated (Opitz et al., 2014). On the other hand, the results of electric field calculations on their own cannot explain the differences in corticomotor threshold, when contrasting stimulation with P-A versus A-P field directions (Kammer et al., 2001). As the induced field distributions are identical for both cases - except for the vectors being mirrored by 180° - the threshold differences are thus exclusively caused by a different impact of the field on the neural elements (Opitz et al., 2014). Without understanding the origin of this effect, strong conclusions on the exact stimulation position and mechanisms in the M1-HAND are premature. Recent realistic simulations started combining accurate field calculations with estimates of how the induced electric field affects the complex neural structures in order to generate more detailed hypotheses on the mechanism of action of TMS. This line of research was started by Salvador et al. (2011) and continued by Pashut et al. (2014), Seo et al. (2016) and Aberra et al. (2020) and will be important to further fine-tune the hypotheses generated by the models. One important consideration is that the outcomes produced by realistic simulations critically depend on which neural elements are included in the simulation and to which extent morphological details of these neural elements are taken into consideration. For instance, the outcome will fundamentally differ depending on how the pyramidal axons are modeled in the simulation (Fig. 2). If the underlying cortical model reduces the pyramidal axon to a simple stick without any branching, the simulation will not be able to show any contribution of the axonal arbor with its axon terminals and collaterals and thus lead to misleading results (Aberra et al., 2020). Therefore, anatomical or physiological insufficiencies of each model should always be fully acknowledged and considered when interpreting and comparing the results of biophysical simulations. Along these lines, future refinements of the biophysical models described above might also test the involvement of superficial sites within the subcortical white matter as possible activation sites for TMS (Laakso et al., 2014). Axons entering white matter would be primarily stimulated at their bends and inverting the current orientation will hyperpolarize a bend that was previously depolarized which may contribute to direction-specific differences in neural excitation for TMS currents in P-A or A-P direction, although this can be accounted for by the activation threshold anisotropy of pyramidal neurons as well. In addition, axonal ramifications in white matter might have low thresholds.

Most TMS studies using MEPs as primary read-out make the implicit assumption that TMS caused a homogenous stimulation of the entire M1-HAND. As discussed in more detail in section 3.2.2, the caudal portion (BA4p) of M1-HAND is located deep in the anterior wall of the precentral sulcus and thus requires higher stimulus intensities for direct targeting. Indeed, systematic comparisons of orientation-dependent MT changes with the electric field changes predicated by biophysical models suggest that the TMS pulse targets primarily the crown or lip of the precentral gyrus (Bungert et al., 2017; Laakso et al., 2018; Weise et al., 2020). In line with the modeling results by Aberra et al. (2020), the absolute strength rather than the normal component of the electric field in that region was found to correlate with neural excitation in two of these studies (Bungert et al., 2017; Weise et al., 2020). This has an important implication for “TMS of the M1-HAND” in the precentral motor hand knob. The PMd in the precentral crown and maybe the very rostral portion of M1-HAND (BA4a) in the posterior lip region of the precentral gyrus are located at the hemispherical surface (Geyer et al., 1996; Geyer et al., 2000) and are therefore primarily stimulated by TMS targeting M1-HAND (see section 3.2).

It is important to point out that there is currently no consensus on which part of M1-HAND is stimulated by TMS. Competing to the hypothesis discussed above that stimulation mainly occurs at positions where the induced electric field strength is highest (i.e., around gyral crowns), it was suggested that it rather occurs where the field component causing an inward current flow perpendicular to the cortical layers is strongest (Chen and Mogul, 2009, 2010; Fox et al., 2004; Salinas et al., 2007, 2009). This hypothesis has been formulated in the *cortical column cosine* theory of TMS efficacy (Fox et al., 2004). Common to all papers on the *cortical column cosine* theory is that the coupling of the electric field on the neurons is modeled in an abstract way, but not derived from detailed biophysical models. The overarching theory states that the coupling of the electric field on cortical neurons will be maximal, if the orientation of the induced E-field is aligned with the main axis of the cortical column (e.g. pyramidal cells) (Arabkheradmand et al., 2018; Krieg et al., 2013, 2015). Given that the field direction in the gyral crown is parallel to the cortical layers, the *cortical column cosine* theory postulates a spatial bias towards stimulation of sulcal positions (Fox et al., 2004). The most superficial part of the gyral wall close to the lip region of the crown constitutes a sweet spot for TMS (Fox et al., 2004). This region is closer to the coil than deeper regions in the sulcal wall, and cortical columns are still optimally aligned to the normal electrical field produced by TMS while being closer to the stimulation coil the coil and (Fig. 2C).

It has been argued that the site of peak activation in the precentral cortex as revealed by ${\text{H}}_{2}^{15}\text{O}-\text{PET}$ studies strongly support the *cortical column cosine* theory (Fox et al., 2004). Using ${\text{H}}_{2}^{15}\text{O}-\text{PET}$ study, it was shown that at train of suprathreshold 3 Hz rTMS of the M1 caused predominantly deep sulcal activity in the posterior part of the M1-HAND in 7 out of 11 participants (Fox et al., 2004). In these 7 participants, the center-of-mass activation was found at x,y,z-coordinates of −32, −32, 48 which was very close to the center-of-mass activation during voluntary movement and corresponds to BA4p where the bulk of the fast-conducting corticospinal output neurons (i.e., the Betz pyramidal cells) are located (Geyer et al., 1996). It should be noted though that a peak activation at x,y,z-coordinates of −32, −32, 48 is located rather deep to be in good agreement with the *cortical column cosine* theory which predicts a peak activation in the superficial part of the sulcal wall (lip region). The lack of activation in the remaining four participants was attributed to juxtacortical axonal excitation, but such activation should also lead to transsynaptic activation of cortical patches created by ortho- and antidromic propagation of action potentials. Other ${\text{H}}_{2}^{15}\text{O}-\text{PET}$, however, also reported more superficial activation spots in the precentral hand knob (Fox et al., 2004; Shitara et al., 2013; Siebner et al., 2001; Takano et al., 2004): Applying sub-threshold rTMS at various rates, ranging from 1–5 Hz, six healthy individuals showed a rate-dependent increase in activity peaking in a more superficial site of the precentral gyrus (x,y,z-coordinates: [C0]38, −22, 56) (Fox et al., 2004; Shitara et al., 2013; Siebner et al., 2001; Takano et al., 2004). Subtracting the effects of afferent stimulation, a more recent TMS-fMRI study demonstrated activation of both the superficial and deeper parts of the M1-HAND by a biphasic suprathreshold TMS pulse (Shitara et al., 2013). In contrast to these PET studies, recent studies have combined functional brain mapping and E-field modeling and provided promising results, pointing to the superficial areas of the crown top and lip region of precentral cortex as primary target sites for TMS (Bungert et al., 2017; Weise et al., 2020).

A note of caution is warranted, when using functional brain mapping of regional neuronal activity to corroborate biophysical models of direct neuronal excitation by TMS. If ${\text{H}}_{2}^{15}\text{O}-\text{PET}$ or fMRI are used to validate models of primary neuronal activation, one should not claim that specific PET or fMRI activations reflect the exact site of direct neural activation by TMS. While modeling work focuses on the induction of action potentials a few milliseconds after TMS, functional activation maps reflect neuronal activity averaged across timescales of seconds (fMRI) or tens of seconds to minutes (PET). Therefore, secondary neuronal effects may dominate the regional neuroimaging read-outs, including transsynaptic spread of excitation and inhibition regionally and at the network level (Siebner et al., 2009). For instance, a ${\text{H}}_{2}^{15}\text{O}-\text{PET}$ study mapped the acute changes in regional cerebral blood flow during and minutes after 150 pulses of 5 Hz rTMS of the left M1 at 90% of active MT (Fox et al., 2004; Shitara et al., 2013; Siebner et al., 2001; Takano et al., 2004). A cluster in the top of the precentral crown showed a well-defined increase in rCBF lasting approximately 8 min after 5 Hz rTMS. peaking at x,y,z-coordinates: −24, −20, 68 which was paralleled by a reduction in intracortical inhibition as evidenced by paired-pulse TMS in a parallel experiment (Fox et al., 2004; Shitara et al., 2013; Siebner et al., 2001; Takano et al., 2004). While these findings indicate a TMS-induced modulation of cortical activity confined to the top of the crown of the precentral hand knob, it does not prove that this spot was the primary site of neuronal stimulation. In conclusion, it is inherently difficult to causally infer the primary site of neuronal excitation from fMRI or PET readouts. Finally, mapping of regional blood flow or regional blood oxygenation levels with PET or MRI provides an integrated readout of net regional neural activity. Hence, a prominent activation of intracortical inhibitory GABA-ergic circuits (deactivation) may counter-balance the activation of glutamatergic pyramidal cells (activation) and thus, obscure the real magnitude of TMS-induced regional activation. This problem has long been recognized in the field of epilepsy research, using EEG-fMRI to capture changes in regional metabolic activity induced by spikes or spike-wave bursts (Gotman et al., 2006).

The *cortical column cosine* theory provides a phenomenological explanation of the directionality effects of TMS. Since large parts of M1-HAND are positioned in the posterior wall of the precentral gyrus (Geyer et al., 1996), the pyramidal cells there are oriented in parallel to the hemispheric surface with their initial axons projecting anteriorly and medially. The theory assumes that these cells are more easily excited than superficial pyramidal cells because the induced currents flow parallel to their main axis and not perpendicular to it. This suggestion was motivated by an early simulation study which observed that a simplified pyramidal neuron with a straight long axon is most effectively depolarized by currents running along the long axis of the neuron and having an orthodromic orientation from the dendritic tree towards soma and axon (Tranchina and Nicholson, 1986). According to this *principle axis effect*, a P-A oriented current direction in the M1-HAND should be optimal to induce direct electrical stimulation of pyramidal cells that have a P-A dendrite-to-axon orientation in the anterior sulcal wall (Laakso et al., 2014). Conversely, an A-P oriented current direction in the M1-HAND should be optimal to induce direct electrical stimulation of pyramidal cells that have an A-P dendrite-to-axon orientation. A limitation is that these predictions are derived from a simplified model of pyramidal cells in which axons are simply straight sticks that have no collateral branches (i.e., lack any axonal arborization). These predictions have not been confirmed in recent modeling studies that either modeled more realistic neural morphologies (Wu et al., 2016) including axonal ramifications (Aberra et al., 2020) or estimated the gyral region directly activated by TMS by combining electric field models with experimental threshold mapping (Bungert et al., 2017; Laakso et al., 2018; Weise et al., 2020) (see also section 2.2). Moreover, the TMS electric field strength decays rapidly with depth and, consequently, even for a simplified neuron model with a “stick” axon, lowest activation thresholds for the anterior bank of the central sulcus are observed in the gyral lip (Fig. 6 in Aberra et al., 2020).

The lack of consensus regarding the primary site of neuronal excitation in human M1 highlights the need for a better understanding of the TMS effects on the level of single neurons and cortical neural circuits. Future progress will depend on further refinements of realistic biophysical models that combine accurate field calculations with estimates of how the induced electric field influences different neurons, combined with systematic tests and comparisons of the predictions of detailed biophysical models with measurements in humans, and direct validation using single cell recordings in animals (e.g., Moliadze et al. (2003); Mueller et al. (2014); Li et al. (2017)). Phenomenological models necessarily stay abstract and allow for less strict tests of the correspondence between model predictions and measurements compared to biophysical models. Phenomenological models should thus be gradually replaced by biophysical models when enough knowledge about cell morphology, channel dynamics, and synaptic properties is available that allow the latter to be implemented well.

### 3.5. Probing cortical excitability of intracortical inhibitory circuits

Since TMS can effectively excite inhibitory interneurons in the cortex, it is possible to study the excitability of intracortical inhibitory networks in M1 using several TMS protocols. The physiological basis for a relatively high susceptibility of intracortical interneurons to TMS-induced excitation is discussed in section 2.4.

#### 3.5.1. Intracortical inhibition at short intervals

A subthreshold conditioning TMS pulse applied at short intervals (1–5 ms) prior to a suprathreshold test stimulus inhibits the test response. This form of inhibition is termed SICI and is dependent on both the intensity of the conditioning and test stimuli (Kujirai et al., 1993). SICI can be obtained with intensities of the conditioning stimulus below active MT and is less expressed when TES is used as either the test or conditioning stimulus (Kujirai et al., 1993). These results provide evidence for a cortical locus for SICI. Additional support for this comes from studies using both single motor unit recordings and epidural spinal recordings (Di Lazzaro et al., 1998c; Hanajima et al., 1998), which provide evidence that SICI involves suppression of late I-waves. The early I1-wave is little affected by the conditioning stimulus and this suggests that SICI does not directly affect pyramidal cell excitability but brings about its inhibitory effect via other intracortical elements. Several mechanisms may contribute to the inhibition at different ISIs. SICI at an ISI of 1 ms may, at least partially, reflect a combination of axonal refractoriness and synaptic inhibition (Fisher et al., 2002; Hanajima et al., 2003) while SICI at an ISI of 2.5 ms (often coinciding with peak inhibition) likely reflects post-synaptic inhibition mediated by GABA_A_ receptors (Ilic et al., 2002; Ziemann et al., 1996b) or, as discussed in more detail in section 2.1, shunting inhibition (Paulus and Rothwell, 2016).

#### 3.5.2. Cortical silent period

The cortical silent period (CSP) describes the relative electromyographic silence seen following the MEP evoked by single-pulse TMS applied during a voluntary contraction. The duration of the CSP increases with stimulus intensity and typically has a maximum duration of 200–300 ms, while -in contrast- the level of voluntary contraction has little influence on the duration (Inghilleri et al., 1993; Kimiskidis et al., 2006; Kimiskidis et al., 2005). Spinal mechanisms contribute to the early part of CSP (~50 ms) (Fuhr et al., 1991), whereas the later part is due to cortical effects (Inghilleri et al., 1993). The duration of the CSP evoked with a TMS pulse is longer than that evoked using a TES pulse (Inghilleri et al., 1993), a finding which provides evidence that the CSP is dependent on activation of an intracortical inhibitory network. Pharmacological evidence suggests that the long-lasting period of inhibition reflects inhibitory cortical activity involving the activation of GABA_B_ receptors (Siebner et al., 1998a; Stetkarova and Kofler, 2013). However, the CSP may also reflect involvement of GABA_A_ receptors at lower stimulus intensities (Kimiskidis et al., 2005; Pierantozzi et al., 2004; Werhahn et al., 1999).

#### 3.5.3. Long-interval intracortical inhibition

Long-interval intracortical inhibition (LICI) is studied by using suprathreshold conditioning and test stimuli applied at ISIs of 50–200 ms. Epidural spinal recordings have shown that the later part of the LICI is associated with suppression of the late I-waves (Chen et al., 1999b). GABA_B_ dependent networks contribute to LICI (McDonnell et al., 2006; Ziemann et al., 2015). These features suggest that LICI and CSP reflect activity of similar networks. However, there is some evidence that the measures are not identical but may have some commonality (Benwell et al., 2007; Hammond and Vallence, 2007). LICI and SICI can interact with SICI being reduced in the presence of LICI (Sanger et al., 2001). Evidence suggests that this is due to a cortical interaction (Ni et al., 2011b) and may be related to presynaptic auto-inhibition mediated by GABA_B_ receptors (Florian et al., 2008). The interactions between different inhibitory networks also highlight the intricate nature of the intra-cortical networks that can be activated and probed using TMS.

### 3.6. Probing the intracortical effects of sensory afferents

All intracortical neuronal elements in M1 that contribute to TMS-induced transsynaptic excitation of fast-conducting cortical output neurons are strongly modulated by afferent activity. Sensory input from the contralateral limb reaches the M1 either through the somatosensory cortex or more directly via thalamo-cortical afferents. Electrical or mechanical stimulation of afferent activity may either depress or enhance the amplitude of MEPs evoked by a single supra-motor threshold TMS pulse (Classen et al., 2000; Tokimura et al., 2000). The modulatory effects of sensory stimulation on MEP amplitude depends on the exact interval (Classen et al., 2000; Tokimura et al., 2000) and the somatotopic relationship between the sensory input and motor output (Dubbioso et al., 2017). At short latencies of around 20 ms, an inhibition (“short-latency afferent inhibition”, SAI) is induced whose origin is cortical (Tokimura et al., 2000). SAI is induced by homotopic stimulation of sensory input that matches the location of the muscle targeted by TMS. Conversely, heterotopic stimulation of a finger distant to the muscle targeted by TMS may produce short-latency afferent facilitation (SAF) (Dubbioso et al., 2017). For homotopic stimulation, MEPs may also be facilitated at slightly longer latencies, whereas at even longer intervals of around 200 ms another afferent inhibition is observed (“long-latency afferent inhibition”, LAI) (Chen et al., 1999a). SAI is mediated by cholinergic and GABA-ergic circuits (Di Lazzaro et al., 2005; Tokimura et al., 2000).

While the effects of homotopic afferent inputs on singe-pulse MEP amplitude are mostly inhibitory (SAI and LAI), afferent inputs induce a disinhibition of local inhibitory circuits (SICI, LICI and the CSP) (Hess et al., 1999; Stefan et al., 2002; Udupa et al., 2009, 2014). The strength of SICF (see section 3.1.1 for details) is facilitated, when being conditioned by electric stimulation of an afferent nerve (Cash et al., 2015). This facilitation increases with increasing strength of SICF or SAI (Cash et al., 2015). LAI also has an inhibitory interaction with other cortical inhibitory circuits such as LICI, but does not appear to influence SICI (Sailer et al., 2002). These examples show that the conditioning effects of afferent somatosensory stimulation on the TMS-evoked MEP amplitude can be readily probed in conditioning-test paradigms, engaging intracortical circuits. These circuits are still incompletely understood and show complex interactions with intracortical circuits that can be assessed with paired-pulse conditioning-test TMS paradigms.

Suprathreshold TMS at rest as used in SAI paradigms induces BOLD activations in the anterior and posterior subregions of M1-HAND (Shitara et al., 2013), corresponding to the location of rostral and caudal sub-divisions (i.e., BA4a and BA4p) found in human cytoarchitectonic studies (Geyer et al., 1996). In the squirrel monkey, the rostral and caudal portions of the M1 receive different afferent input from the limb: the rostral (old) M1 preferentially processes proprioceptive kinesthetic input, whereas the caudal (new) M1 receives mainly cutaneous mechanoreceptive input (Shitara et al., 2013; Strick and Preston, 1982). Therefore, it is likely that the quality of afferent input will have different modulatory effects on cortical circuits in the human BA4a and BA4b. Such differential effects remain to be tested in detail. So far, there is only circumstantial evidence derived from plasticity inducing TMS protocols that two different sets of motor cortical interneurons exist that both contribute to motor output are modulated by afferent activity, presumably receiving input predominantly through a spino-cerebello-thalamo-cortical vs. spino-thalamo-cortical route (Hamada et al., 2014).

In summary, the synchronized stimulation of afferent volleys can be used to study rapid and slow effects on corticomotor excitability as well as the excitability of cortical interneuron circuits in the M1. These effects have mainly been studied in the M1-HAND.

## 4. What do we know about the effects of TMS targeting areas outside the M1-HAND?

### 4.1. Mapping the motor leg representation

Focal TMS with a figure-of-eight coil can be used to selectively target areas in M1 which represent a specific body part because of the somatotopic arrangement in the pericentral sensorimotor cortex. Most TMS studies of M1 targeted M1-HAND, but TMS can also be used to study the cortical representations of other body parts, in particular the representations of the leg or face (Groppa et al., 2012a). The primary motor leg area (M1-LEG) is located at the mesial surface of the M1 in the interhemispheric fissure (Fink et al., 1997; He et al., 1995; Nielsen et al., 1995; Penfield and Rasmussen, 1950). Due to the longer distance between the M1-LEG and the stimulating coil, the leg muscles need higher stimulus intensities for evoking a MEP relative to the M1-HAND. The most frequently studied target muscle in the leg is the tibialis anterior muscle. Because right and left M1-LEG are located close to each other, selective unilateral stimulation is more challenging than for TMS of the hand or face representations located at the hemispherical surface. Descending volleys evoked by both electric and magnetic stimulation over M1-LEG has also been recorded from epidural electrodes in human subjects. It was found that the earliest volley was produced by TES with the anode 2 cm lateral to the vertex whilst the initial volley evoked by TMS occurred 1.1–1.4 ms later. In analogy with the outputs evoked by stimulation of the M1-HAND, these were considered to be a D- and an I1-wave (Di Lazzaro et al., 2001). Another TMS study recorded descending volleys at the level of the thoracic spinal cord elicited by single-pulse TMS of M1-LEG. A similar threshold for eliciting a D- and I1-wave was found in 5 out of 10 healthy individuals, while the I1-wave was recruited first in the remaining 5 individuals (Houlden et al., 1999). Latency measurements from single motor units in the tibialis anterior muscle suggest a predominant activation of I1-waves from M1- LEG at intensities close to MT (Terao et al., 2000). Paired-pulse TMS of M1-LEG has also shown to evoke SICF revealing a short-latency facilitation of MEP amplitude at I-wave periodicity to the same extent as TMS of M1-HAND (Chen and Garg, 2000). Together, these results suggest that TMS of M1-LEG may directly (D-waves) and indirectly (I-waves) activate corticospinal neurons supplying the lower extremities at intensities close to corticomotor threshold.

### 4.2. Can we generalize from M1 to other cortical areas?

The M1 is a unique area, generating direct corticospinal motor output via large fast-conducting pyramidal neurons (giant Betz cells) (Geyer et al., 2000). As pointed out above, transsynaptic excitation of these fast-conducting pyramidal neurons causes a burst of descending volleys in the corticospinal tract which can lead to a MEP. These large pyramidal cells and their corticospinal axons represent a unique output structure that is not present in other cortical areas. The M1 also shows a large amount of myelination and relatively low cell density relative to associative cortical areas. Further, the bulk of M1 does not reach the cortical surface, but is located in the wall of the sulcus (Geyer et al., 2000). Multidimensional scaling analysis of cortical receptor fingerprints revealed “exceptional positions” of motor areas BA4 and BA6, indicating that the microstructure of human precentral cortex differs substantially from most other neocortical areas (Zilles and Palomero-Gallagher, 2017). These neuroanatomical and neurophysiological particularities render it unlikely that knowledge about the neural underpinnings of TMS-induced cortical stimulation gathered with TMS of the precentral gyrus can be transferred one-to-one to cortical areas other than M1.

It is common practice to adjust the intensity of TMS to the individual MT, even when targeting other brain regions. However, it is unlikely that the cortical MT obtained with TMS over the M1 is a reliable indicator for the efficacy of TMS in areas outside M1. Both cortical MTs and phosphene thresholds (PT; subjectively estimated with occipital TMS) allow standardizing intensities in controlled studies to a certain extent. Most studies, however, do not find a correlation when individual PTs are compared with individual resting or active MTs (Antal et al., 2004; Boroojerdi et al., 2002; Stewart et al., 2001), but see (Deblieck et al., 2008). Although MTs are thus most likely an inappropriate guide to the cortical excitability of other non-motor areas of the brain, they are still used due to the lack of efficient alternatives. Individual MRI scans can be used to determine the distance between the coil and the cortical target and this information may be used to adjust the intensity to account for differences in coil–target distance between subjects (Nahas et al., 2004; Stokes et al., 2005). Electric field models based on the MRI scans can further inform individualization of the pulse intensity as well as the coil position and orientation (Beynel et al., 2020; Gomez et al., 2021; Gomez-Tames et al., 2020; Janssen et al., 2014).

### 4.3. Stimulation of cortical areas in the parietal lobe

TMS has contributed to understanding the temporal dynamics of parietal involvement in wide range of tasks probing attention, spatial and sensory-motor processing (Duecker and Sack, 2015; Rushworth and Taylor, 2006). Yet, a systematic investigation of parietal stimulation effects is complicated by large inter-individual variance of the optimal parietal stimulation site for a given task (Ryan et al., 2006). This problem is further emphasized by the fact that the parietal cortex encompasses multiple specialized regions that differ in their functional characteristics. Therefore, TMS studies generally target specific parietal sites according to their scientific question of interest. Knowledge is developing about the effect of parietal stimulation on distant network nodes, indicating remote effects on visual and motor areas (Bestmann et al., 2008; Heinen et al., 2011; Kaneko et al., 2020). Studies also suggest that the effect of parietal stimulation may be highly task-specific and influenced by individual anatomy (Ryan et al., 2006).

While it has been shown that studies benefit from individualized coil placement based on structural or functional MRI (Sack et al., 2009), a consensus for an easy identification procedure of individual stimulation sites is not generally available. In analogy to functional localization of the M1 with the help of the precentral motor hotspot, TMS-induced behavioral interference in visuospatial tasks has been used to functionally determine the individual parietal ‘hotspot’, and at this functional parietal hotspot, individual TMS intensity correlated with the effect of TMS on task-related reaction time (Oliver et al., 2009; Salatino et al., 2014). However, systematic investigations of other parameters such as the optimal coil orientation or pulse number or the impact of task difficulty and specificity remain to be systematically explored.

The effects of parietal stimulation on a network level can be explored independently of behavioural read-outs with dual-site TMS by applying a conditioning TMS pulse at a parietal site and a test pulse at M1 (see section 2.6 for details). An inhibitory parietal influence will decrease MEP amplitude, while a facilitatory influence will have the opposite effect. Using this technique, an innervation gradient along the intraparietal sulcus (IPS) has been found. Conditioning the anterior IPS had an inhibitory effect on M1, whereas conditioning the posterior IPS had a facilitatory parietal-motor effect, with stronger posterior facilitation in the left hemisphere (Karabanov et al., 2013; Koch et al., 2007). These parietal–motor interactions are modulated by task context (Karabanov et al., 2012) and their strength is correlated with individual white matter organization in the parieto-motor tracts (Koch et al., 2010). Parietal-to-M1 interactions are mostly indirect, and mediated by the premotor cortex (Koch et al., 2010). This indirect parietalpremotor-M1 interaction can be studied with a triple-coil technique. Using the influence of the premotor cortex on M1 as readout, a conditioning parietal stimulation can affect premotor-to-M1 interaction at an intensity of stimulation that does not affect MEP amplitude directly (Shields et al., 2016).

Multimodal stimulation studies combining TMS with functional MRI or EEG have further helped to understand network effects outside of M1: TMS-fMRI demonstrated that stimulating IPS produced effects on the BOLD signal in visual area V5/MT+ in a task context and stimulation intensity specific manner (Ruff et al., 2008). EEG recordings of the evoked cortical response revealed distinct region-specific oscillatory ‘signatures’ in response to single-pulse TMS (Fecchio et al., 2017; Rosanova et al., 2009): parasagittal stimulation of the superior parietal lobule evoked oscillations in the beta range (13–20 Hz), while parasagittal stimulation of the frontal or occipital areas evoked gamma and alpha oscillations, respectively (Fecchio et al., 2017; Rosanova et al., 2009). The discovery of area-specific oscillatory signatures which display different frequencies suggests that information processing in these different cortical areas is ‘tuned’ to characteristic frequencies.

### 4.4. Stimulation of visual cortical areas

Visual cortex comprises posterior cortical regions V1–V6 and has been targeted by TMS to clarify the working mechanisms of TMS or to investigate the functional specialization of specific visual areas. The latter category of studies used focal TMS to temporarily disrupt functional processing in the cortical target region. This interference approach proved to be useful to demonstrate and characterize causal links between functional specialization of visual cortical areas and visual perception. Its temporal resolution enabled to assess the temporal flow of information between functionally or anatomically connected visual areas. Seminal studies in the field confirmed that TMS over early visual areas V1/V2 in the occipital lobe can impair visual perception of shortly presented three-letter stimuli at a specific time window, peaking 70–100 ms after the presentation of the visual stimulus (Amassian et al., 1989; Beckers and Homberg, 1992; Matthews et al., 2001; McKeefry et al., 2008). Later it was shown that TMS of areas V1/V2 suppresses the perception of low-contrast achromatic stimuli activating the magnocellular visual pathway already at an earlier time window (i.e., 40 ms earlier) than low-contrast chromatic stimuli activating the parvocellular pathway (Paulus et al., 1999a). Focal TMS targeting area V5 at the border of the temporal and occipital lobe has been used to weaken visual motion perception, modify direction discrimination and speed acuity (Amassian et al., 1989; Beckers and Homberg, 1992; Matthews et al., 2001; McKeefry et al., 2008).

TMS over the visual cortex can not only disrupt perception but can also induce a transient perception of a flash of light, referred to as phosphenes. Phosphenes elicited by TMS of V1/V2 are small and static, whereas those induced by TMS over V5 are large, reflecting the cortical magnification factor, and are moving (Kammer et al., 2005a; Silvanto and Muggleton, 2008). Early studies (e.g. Kammer (1999)) have demonstrated a relatively good topographic correspondence between TMS-induced phosphenes and TMS-induced visual field deficits (scotomas) induced from several stimulation sites, suggesting that phosphenes could aid as a guide for visual stimulus alignment in psychophysical studies. Both TMS-induced phosphenes and scotomas can be elicited from a large area over the occipital lobe, with a large range of possible coil positions. However, the stimulation intensities needed to induce phosphenes are lower than the TMS intensities needed for inducing scotomas. They correspond mainly to the stimulated hemisphere and, when stimulating over V1/V2, occur mostly in the lower visual field, consistent with a stimulation of the dorsal parts of the early visual areas. The core of the scotomas matches to the core of the induced phosphenes. It is worth pointing out that certain characteristics of TMS-induced phosphenes, such as the texture, shape and position in the visual field are stable when the coil is positioned at different scalp positions overlying early visual cortical areas in the same hemisphere (Salminen-Vaparanta et al., 2013). One may argue that this constancy indicates that TMS-evoked phosphenes cannot be related exclusively to one functionally defined visual area. Alternatively, the finding that TMS elicits stable percepts that are relatively independent of the coil position may indicate that the same neuroanatomical structure is always stimulated, for instance the same occipital gyrus or portion of the optic radiation (Marg and Rudiak, 1994).

Complementing the MT derived by TMS targeting the precentral cortex, the phosphene threshold (PT) has been used to probe visual cortical excitability alone or in combination with other methods, e.g., with EEG or fMRI (e.g., Schwarzkopf et al. (2010); Silvanto et al. (2010)). Applying a probability criterion, the PT is most frequently defined as the intensity of TMS at which the observer reports phosphenes on 50% of trials. It was proposed that the causal relationship between cortical excitation and phosphene perception depends on the power and phase of the ongoing occipital alpha activity (Dugue et al., 2011; Romei et al., 2008). Cortical stimulation, occurring between the peak (phase = 0) and the next zero-crossing (phase = π/2) of the occipital alpha oscillation, is most likely to produce phosphenes (Dugue et al., 2011). Nevertheless, as it was mentioned above, the generation of phosphenes cannot exclusively be related to a certain functionally defined visual area. It was suggested that both the optic radiation close to its termination in the dorsal parts of V1 and back-projecting fibers from V2 and V3 back to V1 could generate visual percepts (phosphenes) and deficits (scotomas) in distinct parts of the visual field (Kammer et al., 2005a; Thielscher et al., 2010).

Studies in animals have revealed important insights into the neurophysiological mechanisms of action of TMS by targeting visual cortical areas (see section 2.4). In the visual cortex of the cat, single biphasic TMS pulse applied over the occipital pole with an electric current flowing from lateral to medial elicited neuronal facilitation for 500–1000 ms, followed by synchronous suppression of activity lasting up to a few seconds (Moliadze et al., 2003) or minutes (Allen et al., 2007), affecting a large pool of neurons (Kozyrev et al., 2014). The immediate effect of the stimulation could be related to a direct or indirect threshold-dependent stimulation of inhibitory and excitatory interneurons, e.g. by inducing fast intracortical inhibition (Kozyrev et al., 2014), and/or by disrupting the temporal structure of activity by altering phase relationship between neural signals (Allen et al., 2007). Modeling studies support these observations in a local circuit model: a single-pulse TMS within a limited time window after the sensory afferent input suppressed spiking activity and disrupted the population response (Miyawaki et al., 2012). The magnitude of suppression was significantly larger for synaptically-connected neurons than for isolated neurons, suggesting that intracortical inhibitory synaptic coupling plays a role in the induced suppression. The suppression phase can be blocked by a 10 Hz train of rTMS (Kozyrev et al., 2014), as was revealed by optical imaging with voltage-sensitive dye in the visual cortex of cats. Other studies found that a single pulse or trains of TMS affect the activity not only of the visual cortical neurons but also those in the large cortico-geniculate feedback pathway, connecting V1 and the dorsal lateral geniculate nucleus (de Labra et al., 2007). It was hypothesized that this involves inactivation of the cortico-geniculate downflow, affecting mainly the tonic neuronal activity (de Labra et al., 2007).

Many of these findings in animals have implications for the application of TMS to study the visual system in humans. For instance, measurements of spiking activity and local field potentials in the cat visual cortex suggested that TMS has state-dependent effects: higher pre-TMS activity predicted larger post-TMS responses (Pasley et al., 2009). In human studies, it was also observed that the neural impact of a stimulation is determined not only by stimulus properties, but also by the baseline activation state of the targeted brain region: TMS impairs motion detection ability when it is applied over V5 during a simple motion detection task, but using the same stimulation parameters it facilitates motion detection if the targeted area was experimentally suppressed prior to the task (Cattaneo and Silvanto, 2008; Silvanto et al., 2008; Silvanto and Pascual-Leone, 2008). Exploitation of visual adaptation led to the observation that the less active visual neuronal populations within the targeted area react stronger to TMS, suggesting that the sensitivity of cortical neurons to TMS depends on their on-going firing rates. This reduces the signal-to-noise ratio and results in a behavioral disruption. However, in this effect the interaction between the stimulus strength and the TMS intensity is critical, by decreasing the TMS intensity the visual performance can be increased (Abrahamyan et al., 2011; Schwarzkopf et al., 2011). This low-intensity phenomenon may reflect stochastic resonance introduced by low-intensity TMS, which enhances information transfer by the addition of low levels of noise (Miniussi et al., 2013; Miniussi et al., 2010). This, in turn, lowers the threshold for visual perception at the behavioral level.

Developments in the field during the last decade imply that the application of rhythmic TMS is a promising tool to entrain cortical activity (Herring et al., 2015; Thut et al., 2003; Thut et al., 2011). It was suggested that in the visual cortex, TMS-evoked oscillations are generated by the same neuronal circuits as the targeted spontaneous oscillations (Herring et al., 2015). Oscillations in the alpha-frequency band evoked by single-pulse TMS were modulated by top-down attention in the same direction as spontaneous alpha oscillations, increasing in amplitude when visual attention was low and decreasing when it was high. Therefore, rhythmic TMS can be an effective tool to study the causal role of neuronal oscillations in visual perception.

### 4.5. Stimulation of prefrontal cortex

TMS to the prefrontal cortex (PFC) has helped to establish the role of the PFC in cognitive functions like memory (Rossi et al., 2012), language (Cattaneo, 2013) and decision making, as well as the internal milieu of behavioral motivations and emotions (Levasseur-Moreau and Fecteau, 2012; Mondino et al., 2015) in a deterministic framework (Miniussi et al., 2013). All these depend on the ability of TMS to transiently interact with the activity of specialized functional networks, especially when applied “online” during task execution. The immediacy of TMS-induced online effects can establish a clear relationship between cause and effect, if not directly then at least through a chain of intermediate mechanisms (Bergmann and Hartwigsen, 2021). For M1, it is well known that the threshold, latency, and amplitude of the MEP response evoked by TMS strongly depend on the direction of the induced current in the precentral gyrus (see section 2.1). A behavioral TMS study showed that the orientation of the induced electrical current is also relevant when stimulating the prefrontal cortex (Hill et al., 2000). TMS was applied with a figure-of-eight coil, placed at one of eight different orientations over the dorsomedial prefrontal cortex, while participants performed a memory-guided saccade task. It was found that the most effective current orientation to interfere with memory-guided saccades was antero-lateral (Hill et al., 2000). This direction-specific behavioral effect indicates that the induced current direction in the cortical target area needs to be considered as independent variable, when studying online effects of TMS on task performance.

The effects of prefrontal TMS are not only mediated by TMS-induced changes in the PFC region that is directly stimulated by TMS. Given the complex interactions of the PFC with other areas, TMS affects the functional interaction of the stimulated area with connected brain areas, which may contribute substantially to the effects of prefrontal TMS at the behavioral level. These connectivity-based network effects should not be viewed as a limitation. Methodological control conditions achieved by stimulation of cortical areas or by looking at time course of interactions between nodes will reveal distinct specializations. For example, it might be possible to establish functional connectivity and a hierarchy of control (i.e., from the PFC to other cortices) by means of multi-site TMS or TMS in combination with neuroimaging procedures (Castrillon et al., 2020; Lorenc et al., 2015; Siebner et al., 2009). For instance, a conditioning TMS pulse of the pre-supplementary motor area in the medial prefrontal cortex facilitated MEPs elicited by a TMS test pulse over M1-HAND, when actions had to be re-programmed (Mars et al., 2009). Dual-site TMS did not produce any MEP facilitation, when the same actions had to be produced in the absence of conflict.

Prefrontal TMS has also been used to characterize the differential involvement of the same cortical area in different cognitive tasks. Here, the same protocol may induce opposite effects based on the specific cortical engagement and capacities related to the cognitive function that are engaged by the experimental task. For example, the same TMS protocol applied to the same PFC region may facilitate a cognitive function, e.g., action naming (Cappa et al., 2002), yet impair long-term memory (Rossi et al., 2001). Therefore, behavioral effects induced by prefrontal TMS require a nuanced interpretation that considers the specific task context and generalizing conclusions should be made with caution.

Prefrontal TMS is often personalized by adjusting the intensity to individual resting motor threshold as determined at M1-HAND, but, as discussed above, this dosing procedure relies on the assumption that prefrontal cortex and precentral gyrus are equally responsive to TMS. Kahkonen et al. (2004) used the TMS evoked cortical potential to capture the dose–response relationship of the prefrontal cortex. Applying single TMS pulses to dorsomedial prefrontal cortex, they found that the TEPs at the Fz/FCz electrodes scaled positively with stimulus intensity (Kahkonen et al., 2004). This TEP response was already observed at 60% of the MT (assessed at M1-HAND), and TEP amplitude increased as stimulation intensity is increased (Kahkonen et al., 2005). While these data suggest that M1 threshold may be used for determining the TMS intensity of PFC (see also Kaminski et al. (2011)), a recent study by Conde et al. (2019) was able to produce very similar TEPs as those that had been published by Kahkonen et al. (2005) with a realistic sham stimulation. The realistic sham TMS procedure mimicked the auditory and somatosensory co-stimulation in the absence of significant transcranial stimulation of the prefrontal cortex (Conde et al., 2019). Together, these studies indicate that the regional TEP response to prefrontal TMS may be used for individual dose adjustment, but this procedure is only valid if the procedure controls for peripheral co-stimulation.

### 4.6. Stimulation of premotor areas in the frontal lobe

Premotor areas are located rostral to M1 and thus can be targeted using the precentral motor hot spots as reference points. The premotor cortex has traditionally been functionally divided into dorsal (PMd) and ventral (PMv) premotor areas. Recent studies however suggest a more complex organization, e.g. that PMd itself is composed of several functional subdivisions (Genon et al., 2017; Genon et al., 2018). The PMd and PMv are located anterior to the hand and face representations of the M1. Since the PMd and PMv are located at the hemispheric surface, these regions can be targeted with TMS using relatively low stimulus intensities. The caudal supplementary motor area (also referred to as SMA-proper) is located medially within the interhemispheric fissure just in front of M1-LEG. Due to its rather deep location relative to the hemispheric surface, effective stimulation of the SMA using TMS requires relatively higher stimulation intensities than PMd and PMv and will also result in concurrent stimulation of more superficial and medial parts of the PMd. Caudal premotor areas can indirectly influence corticospinal motor output via dense cortico-cortical connections to M1, but also potentially directly via descending, di-synaptic projections to spinal motoneurons (Dum and Strick, 2005).

Most TMS studies have typically investigated the role of PMd and PMv in the context of skilled hand movements. It has been shown that PMd contributes to the anticipatory control of grasping movements when they are conditioned to external cues, such as arbitrary associations between color and object weight (Chouinard et al., 2005; van Nuenen et al., 2012), but also to internal sensorimotor signals, such as lift initiation after enough tactile information has been accumulated (Davare et al., 2006; Loh et al., 2010). These TMS findings fit well with recordings in non-human primates showing that PMd (F2) contains a hand representation (Raos et al., 2004). Recordings of neural activity in this area identified neurons which exhibit cue-specific preparatory activity (Cisek and Kalaska, 2005). It is therefore plausible that TMS interfered with the firing rate of this neuronal population, hence leading to measurable deficits in grasp control. Further, dual-site TMS experiments probed PMd-to-M1 interactions and revealed short-latency (1.2 ms) net facilitatory effects of ipsilateral PMd on corticospinal output from M1-HAND (Groppa et al., 2012c). The expression of this ultra-short ipsilateral premotor-to-motor facilitation was modulated by task context, depending on the cued motor response (Groppa et al., 2012c). Hence, dual-site TMS can probe how preparatory motor activity encoded in PMd contributes to motor output generated in M1 during cued motor tasks.

TMS applied over PMv was shown to disrupt hand shaping and alter the pattern of hand muscle recruitment when grasping an object (Davare et al., 2006). Interestingly, neuronal recordings in the monkey PMv (F5) demonstrated the existence of visuomotor ‘canonical’ neurons whose firing rate was grasp- and object shape-specific (Rizzolatti and Luppino, 2001). Based on these two findings, Davare et al. used a dual coil TMS protocol where a conditioning pulse over PMv probed the effect of underlying neural populations on a test pulse applied 6–8 ms later over M1 (Davare et al., 2008). Thus, the rationale was to experimentally manipulate the excitability of visuomotor neurons in PMv by asking subjects to perform a precision grip vs. a whole hand grasp, which would in turn show grasp-specific effects of PMv conditioning on M1 output. At rest, PMv conditioning exerted a net inhibitory influence on M1, whereas during grasp preparation PMv facilitated M1 in a muscle- and grasp-specific fashion (Davare et al., 2009; Davare et al., 2010). Interestingly, this demonstrates that the susceptibility of a given neural population to TMS varies based on its excitability level. A similar physiological mechanism can also explain the fact that net inhibitory or facilitatory PMv-M1 interactions can be found by varying the intensity of PMv-TMS conditioning, an experimental manipulation likely to recruit different PMv output neurons with different connectivity profiles (Baumer et al., 2009). In line with this view, another study found that right PMv-left M1 physiological interactions switch from facilitation to inhibition when the grasp had to be corrected online (Buch et al., 2010). Again, the mechanism underlying this effect can be explained if the implementation of corrective grasp motor commands brings into play another neural population with a prominent inhibitory influence on M1.

### 4.7. Stimulation of the cerebellum

One major challenge for quantifying the effects of cerebellar stimulation is the lack of any direct, non-invasively measurable output. The effects can only be inferred from secondary measurements, either physiological or behavioural. Physiological measurements allow for evaluation of both instantaneous (online effects) or delayed consequences (after-effects) of cerebellar TMS, whereas behavioural read-outs are only suited (with few exceptions) to evaluate after-effects of cerebellar TMS. Two physiological approaches to probe the impact of cerebellar TMS stand out so far, as they offer excellent temporal resolution: one approach measures modulatory effects of cerebellar TMS on corticomotor excitability in M1 (Daskalakis et al., 2004), while the other uses eye movement recordings as psychophysiological read-out (Colnaghi et al., 2010). To explore these discrete cerebellar-motor interactions in humans, cerebellar output can be manipulated non-invasively either with electrical or magnetic pulses (Ugawa et al., 1991; Ugawa et al., 1994; Ugawa et al., 1997; Ugawa et al., 1995).

Despite their ideal temporal resolution, there is one major limiting factor: the scalp-to-cerebellar cortex distance is more than three times longer and more variable than the distance from scalp to M1-HAND. This leads to poor focality and neuronal population targeting (Wagner et al., 2009; Wagner et al., 2007). Indeed, some authors have found that smaller coils are unable to activate cerebellum because their fields do not penetrate sufficiently deep below the scalp (Spampinato et al., 2020). Most authors use large coils (such as the double cone coil) for cerebellar stimulation, and further improvements might be achieved with non-focal TMS coil designs with higher depth penetration (Hardwick et al., 2014). However, such solutions raise further problems. These coils are less comfortable because they produce strong neck muscle contraction and activate afferent axons in nerves of the cervicobrachial plexus (Werhahn et al., 1996). Non-focal stimulation may also activate the neighbouring occipital lobe and result in anti- and orthodromic co-activation of the large myelinated axons in the corticospinal tract or other major ascending fibre tracts in the brain stem. So far, two methods have been proposed to address these problems: one is to adjust the stimulation intensity according to the individual distance between the scalp and the cerebellar target (Popa et al., 2010), while the other is to identify the intensity required to activate pyramidal tract axons in the brainstem beneath the cerebellum. Cerebellar stimulation limited to intensities lower than this threshold would reduce brainstem activation and have the additional advantage of avoiding antidromic conduction of pyramidal tract impulses back into M1 (Fernandez et al., 2018; Ugawa et al., 1995).

At the same time, the critical role of the distance between the coil and the cerebellar target also represents an advantage for applying an ideal, active sham stimulation. The active sham condition is represented by a real stimulation delivered with the exact same intensity as in the real TMS condition, but with the coil placed 5 cm below the cerebellar target on the back of the neck (Kishore et al., 2014; Popa et al., 2010). This realistic sham TMS condition induces the same twitch in the neck muscles, produces the same sound, and stimulates similarly the brachial plexus, but without delivering a neurobiological significant stimulation to the cerebellar target.

A better understanding of what structures within the cerebellum are responding to TMS could also help us adjust the stimulation intensity and orientation appropriately. A few studies have addressed this problem by modelling the effective electric fields that are induced by TMS in the neuronal structures of the posterior fossa (Bijsterbosch et al., 2012; Guadagnin et al., 2014; Hardwick et al., 2014; Rampersad et al., 2014). They concluded that the effects critically depend on the subjacent anatomy, tissue composition, and coil placement - individual modeling being necessary for minimizing outcome variability. Regarding the modeling of the electric fields in the cerebellar folia, it is still unclear whether they show a similar directional dependency as shown for the cortical gyri of the neocortex. In the cerebrum, direction-specific effects depend on the cerebrospinal fluid compartment on both sides of the gyral crown, but the situation may be very different in the cerebellum with its densely packed folia (Opitz et al., 2011). It is still unclear which neural structures in the cerebellar cortex are most susceptible to stimulation and how much the underlying white-matter fibers can be effectively stimulated. Are the Purkinje cells stimulated directly or transsynaptically through the parallel or climbing fibers? Animal models, along with realistic individual head models and field distribution for humans are needed to answer these questions. However, despite of the uncertainty regarding its spatial resolution and target, the superb temporal resolution of the TMS pulses bears great potential for empirical studies of cerebellar function, especially when exploring physiological effects.

Two experimental approaches can currently provide clues regarding the structures that are stimulated within the cerebellum. One approach employs dual-site conditioning-test TMS targeting the cerebellar hemisphere and the contralateral M1-HAND. The second approach involves single TMS pulses delivered over the ocular motor vermis (posterior vermis, lobule VIc and VII) during saccade adaptation. For dual-site cerebellar-cortical TMS, the first coil is placed over the cerebellum and applies a conditioning stimulus to the posterior cerebellum. This conditioning cerebellar stimulation can reduce the amplitude of MEPs evoked by a test pulse delivered over M1-HAND 5–7 ms after the cerebellar conditioning stimulus (Ugawa et al., 1995). The effect is due to activity in cerebellar outflow fibres in the superior peduncle which conduct impulses to cortex via thalamus and is absent in patients with ataxia with lesions within this pathway (summarised in Iwata and Ugawa (2005)). The final projections of the cerebellothalamo-cortical pathways also seem to project onto cortical interneurons, at least in M1-HAND, since cerebellar conditioning pulses decrease not only MEPs but also SICI, while facilitating ICF (Brighina et al., 2009). Moreover, continuous theta-burst stimulation (cTBS) of the cerebellum reduces SICI and increases LICI, while cerebellar iTBS reduces LICI (Koch et al., 2008).

The conditioning-test approach is most frequently referred to as cerebellar-brain inhibition (CBI) or less commonly, but more specifically, cerebello-motorcortical inhibition (Ni et al., 2010). This cerebello-motorcortical inhibition can be temporarily suppressed by low-intensity 1 Hz rTMS or cTBS delivered to the cerebellum with a flat coil that cannot reach deeper than the cerebellar cortex (Popa et al., 2010), or by tDCS that induces a local change of regional excitability in the cerebellar cortex (Batsikadze et al., 2019). It was hypothesized that cerebellar TMS pulses activate the Purkinje cells, which send inhibitory projections onto the den-tate nucleus, thus resulting in a de-facilitation of the tonically excitatory dentato-thalamo-cortical pathway (Daskalakis et al., 2004; Pinto and Chen, 2001; Ugawa et al., 1997; Ugawa et al., 1995). Recent electrophysiologic measurements in animals support this view: superior cerebellar peduncle stimulation in monkeys evoked motor cortical responses at a latency consistent with a bi-synaptic projection (Nashef et al., 2018). Yet, does the cortico-to-nuclear de-facilitation hypothesis account for the temporal profile of cerebello-motocortical inhibition? The cerebellar TMS pulses elicit a suppression of MEP amplitude already after a few milliseconds. Such short-latency onset inhibition can be evoked with paired-pulse TMS of M1-HAND and reflects the temporal dynamics of inhibitory postsynaptic potentials (i.e., SICI). Even if the TMS pulse acutely blocks the output from the excitatory dentato-thalamo-cortical pathway, it is unlikely that this will immediately and synchronously remove all the excitatory action potentials that are on their way to the cortex. Since this excitatory drive is temporally dispersed, its removal should take at least a few milliseconds. Therefore, one might expect a later onset of CBI, if cortico-to-nuclear de-facilitation is the key mechanism.

Another approach employs single TMS to target the ocular motor vermis (posterior vermis, lobule VIc and VII) during saccade adaptation. TMS of these structures can induce hypometric contralateral and hypermetric ipsilateral saccades (Hashimoto and Ohtsuka, 1995), accelerate ipsiversive pursuit and decelerate contraversive pursuit (Ohtsuka and Enoki, 1998), and reduce the delay between eye and head movements in coordinated eye-head movement tasks (Nagel and Zangemeister, 2003). These effects were attributed to the TMS pulse increasing the inhibitory output from the paravermal Purkinje cells to the ipsilateral fastigial nucleus, thus introducing an imbalance in the control of the pontine and midbrain burst neurons (Colnaghi et al., 2010).

Evidence for the influence of cerebellar TMS on non-motor cortical areas in humans is still indirect, coming mostly from fMRI studies. Both intermittent and cTBS of the cerebellum appear to modulate the functional connectivity between the cerebellum and the default mode network, dorsal attention system, and frontal and parietal cognitive regions (Halko et al., 2014; Rastogi et al., 2017). More indirect support for the effects of cerebellar stimulation on non-motor functions begin to emerge from cognitive neuroimaging studies (e.g., verbal working memory (Sheu et al., 2019), short term memory of visual sequences (Ferrari et al., 2018a), perception of emotional content (Ferrari et al., 2018b), attentional control (Esterman et al., 2017), visuomotor learning adaptation (Koch et al., 2020), or sequence learning: (Ballard et al., 2019), etc.).

## 5. Concluding remarks

More than three decades have passed since TMS was introduced as a non-invasive method to stimulate the human cortex. Although it has undoubtedly pushed the frontiers of human neuroscience and interventional neurophysiology with a potential for therapeutic applications, we still lack a comprehensive understanding of which neural elements in the cortex are primarily targeted with TMS. It is fair to conclude that TMS induces action potentials by depolarizing myelinated axonal structures in the stimulated cortex and that this regional excitation may spread via cortico-cortical and cortico-subcortical connections to connected brain regions. Both the regional as well as remote effects due to spread of excitation via neural fibers are highly state-dependent and involve excitatory (glutamatergic) and inhibitory (GABA-ergic) neurons. More detailed statements regarding the preferential site of excitation (e.g., gyral crown versus sulcal wall) or the sensitivity of distinct classes of intracortical neurons are still debated, although some progress has been made in recent years to clarify these questions. More basic research on the biophysics and neurobiology of TMS is needed to get further basic insight into its mechanisms of action. Preclinical research in animals, particularly recordings from single cells and the use of advanced methods such as optical imaging and optogenetics in combination with neuroimaging and biophysical modeling need to work hand in hand to clarify how TMS interacts with the human brain. The complexity of the brain’s response to TMS provides a challenge for the interpretation of its (neuro)physiological and behavioural effects (Bestmann and Krakauer, 2015), and cautions against simplistic rationales for its application. A deeper understanding of how a magnetic pulse (or pulses) stimulates the brain from single cell types to microcircuits to brain networks is a prerequisite for linking the physiological and behavioural consequences of TMS, tailoring TMS to individual brains and for advancing it as a scientific and therapeutic tool.

## HIGHLIGHTS

- TMS primarily targets the gyri at the hemispheric surface due to limited depth penetration.
- The direct response to TMS is complex, involving a mixture of neuronal populations.
- Myelinated axon terminals of pyramidal cells and inhibitory interneurons in the crown of the gyri constitute low-threshold targets for TMS.
- Neuronal excitation propagates along axons and across synapses from the primary stimulation site to connected regions in a state-dependent fashion.
- TMS always causes substantial peripheral somatosensory and auditory co-stimulation.

## Abbreviations:

A-P: Anterior-to-posterior
BEM: Boundary element method
CBI: Cerebellar-brain inhibition
CSP: Cortical silent period
cTBS: Continuous theta-burst stimulation
D-wave: Direct wave
EEG: Electroencephalography
FEM: Finite Element Methods
fMRI: Functional magnetic resonance imaging
GABA: c-aminobutyric acid
I-waves: Indirect waves
IPS: Intraparietal sulcus
ISI: Interstimulus interval
iTBS: Intermittent theta-burst stimulation
L-M: Lateral-to-medial
LAI: Long-latency afferent inhibition
LICI: Long-interval intracortical inhibition
M1: Primary motor cortex
M1-HAND: Hand representation of primary motor cortex
MEP: Motor evoked potential
MT: Motor threshold
P-A: Posterior-to-anterior
PAS: Paired associative stimulation
PET: Positron emission tomography
PFC: Prefrontal cortex
PMd: Dorsal premotor cortex
PMv: Ventral premotor cortex
PT: Phosphene threshold
rCBF: Regional cerebral blood flow
rCMRglu: Regional metabolic rate of glucose
rTMS: Repetitive transcranial magnetic stimulation
S-D: Strength–duration
SAF: Short-latency afferent facilitation
SAI: Short-latency afferent inhibition
SICF: Short-interval intracortical facilitation
SICI: Short-interval intracortical inhibition
TEP: TMS evoked EEG potential
TES: Transcranial electric stimulation
TMS: Transcranial magnetic stimulation
VGSC: Voltage-gated sodium channel Indices
ANT: anterior
POST: posterior.
